# Do methane emissions converge? Evidence from global panel data on production- and consumption-based emissions

**DOI:** 10.1007/s00181-021-02162-9

**Published:** 2021-12-09

**Authors:** Octavio Fernández-Amador, Doris A. Oberdabernig, Patrick Tomberger

**Affiliations:** 1 World Trade Institute, University of Bern, Hallerstrasse 6, 3012 Bern, Switzerland; 2 University of Innsbruck, Universitätsstraße 15, 6020 Innsbruck, Austria

**Keywords:** Methane emissions, Beta convergence, Emission footprints, Emission intensities, Sectoral analysis, F64, O44, Q54, Q56

## Abstract

Methane emissions are the second most important contributor to global warming. Knowledge about the dynamics of methane emissions facilitates the formulation of climate policies and the understanding of their consequences. We investigate whether methane emissions released from production and embodied in consumption converge within and across regions. Our estimates rely on global panel data on methane per capita and methane intensities over 1997–2014. We find that emissions converge within countries. The short half-lives show that the emissions of countries are close to their steady states. There is no evidence for international convergence of aggregate emissions. Yet, convergence of emissions across regions occurs in a number of economic sectors. Our results highlight the difficulties to achieve methane abatement in the medium run. The formulation of climate policies should take into account the sectoral specificity of the dynamics of methane emissions.

**Supplementary Information:**

The online version supplementary material available at 10.1007/s00181-021-02162-9.

## Introduction

The Paris Agreement delegates to national governments the formulation of greenhouse gas (GHG) abatement targets, implemented through Nationally Determined Contributions (NDCs; Jacquet and Jamieson 2016; Nieto et al. 2018; Keohane and Victor 2016). This bottom-up approach to emission abatement calls for a better understanding of national GHG emissions trajectories (see also Joeri Rogelj 2016). The debate on GHG emissions so far has mainly focused on carbon dioxide (CO$_2$) emissions, whereas much less attention has been paid to the second most important GHG, methane (CH$_4$).

Global anthropogenic CH$_4$ releases are comparable in magnitude to CO$_2$ emissions in terms of their global warming potential (GWP) over a period of 20 years (Fernández-Amador et al. 2020b). Yet, CH$_4$ emissions differ from CO$_2$ emissions in important aspects. CH$_4$ has a much shorter atmospheric lifetime than CO$_2$ and a considerably larger GWP per ton of emissions, which is concentrated at the beginning of its atmospheric life (IPCC 2014). In contrast to CO$_2$, the bulk of anthropogenic CH$_4$ emissions is attributed to few economic activities with very heterogeneous abatement potentials and costs, such as cattle breeding, drilling and transportation of fossil fuels, waste management, and rice cultivation. This impacts on the geographic distribution of emissions, with developing countries contributing a major part to global CH$_4$ releases (Fernández-Amador et al. 2020b). Also, the determinants of CH$_4$ emissions differ from those of CO$_2$ emissions, notably the effect of economic growth and of income per capita (Fernández-Amador et al. 2018, 2020a). These differences, and the problems connected to the conversion of different GHGs to a common scale (Fesenfeld et al. 2018), have been one of the stumbling blocks for negotiations about the rules for international GHG markets under Article 6 of the Paris Agreement, at the 25$^{th}$ Conference of the Parties (COP25) in Madrid (Carbon Brief 2019). Because mitigation of CH$_4$ emissions is especially important for limiting climate change in the near term (Jackson 2009; Shindell et al. 2012, 2017), more attention to CH$_4$ emissions is warranted.

Knowledge about convergence dynamics of CH$_4$ emissions is important in at least three aspects: it allows to evaluate equity concerns about the effects of climate policy, to better understand the costs related to such policy, and to provide information for climate models. If emissions converged internationally, equity concerns related to international climate policy aimed at changing the distribution of emissions across countries could be reduced (see Aldy 2006; Barassi et al. 2011). By contrast, the absence of international convergence could point to persistent structural differences across countries, such that international climate policy would introduce substantial reallocation of emissions (Aldy 2006). Thus, testing for international convergence of emissions is relevant to understand the distributional dynamics of emissions and countries’ participation and position in multilateral agreements.

Furthermore, the focus on NDCs inherent in the Paris Agreement emphasizes the importance of country-specific convergence dynamics. Country-specific convergence provides information about how far a country’s emissions are from their equilibrium (steady-state) value. This information allows to assess emissions in relation to a country’s long-run economic growth and to determine its long-run national contribution. This information is also relevant to evaluate the costs of emission reduction targets. When a country is close to its equilibrium path of emissions and that path has revealed to be unsustainable, a large share of emission reduction will be associated with the equilibrium path. By contrast, when a country is far from its steady state, a larger share of emission reduction will be associated with the transition dynamics. In general, for a given level of emission reduction, the costs of abatement will be larger if countries are closer to their steady-state level of emissions.

Moreover, the hypothesis of convergence usually underlies projections of climate models that are used to evaluate the effects of climate policy (Romero-Ávila 2008; Barassi et al. 2011). Empirical studies on the convergence of GHG emissions can provide evidence whether this assumption is justified.

The empirical literature has tested for convergence of GHG emissions per capita and per unit of value added (emission intensity), mainly focusing on CO$_2$ emissions (Pettersson et al. 2014; Stern 2017). Although most studies found evidence for convergence or convergence clubs for CO$_2$ per capita, some rejected the existence of convergence.^1^ Some countries, such as China and India, have defined their NDCs in terms of emission intensities. Emission intensities can be more directly addressed by national environmental policies than emissions per capita, because the economic scale of production, affecting emissions per capita, is determined not only by local but also by global demand. Thus, it is informative to assess whether missing convergence of emissions per capita is driven by missing convergence of emission intensities. If emission intensities do not converge, policies aimed at the adoption of less emission intensive production methods may contribute to emission abatement, especially in developing countries that have higher emission intensities. Empirical studies on the convergence of CO$_2$ intensities detected evidence for convergence (Camarero et al. 2013; Panopoulou and Pantelidis 2009; Fernández-Amador et al. 2019).

Most empirical studies have focused on production-based emissions, because of the political emphasis on territorial emissions to determine responsibility for emissions;^2^ however, the interest in consumption-based emissions is rapidly increasing.^3^ Consumption-based emission footprints assign the responsibility of emissions released in the production of products to the country in which final consumption takes place, and thus account for the spatial separation of production and consumption in a globalized world. Hence, convergence in emissions embodied in consumption per capita may address equity concerns even more directly than convergence of production-based emissions per capita (see Lenzen et al. 2012). Convergence of CO$_2$ embodied in consumption across countries has been evaluated by Fernández-Amador et al. (2019) and Karakaya et al. (2019), who found evidence for convergence.

In this paper, we provide a comprehensive analysis of convergence patterns of CH$_4$ emissions. Our estimates are based on global panel data on anthropogenic CH$_4$ emissions for 66 countries and 12 composite regions in the period from 1997 to 2014, available from Fernández-Amador et al. (2020b). Our analysis contributes to the literature in several ways. First, we assess convergence of CH$_4$ emissions within regions, toward individual-specific steady states, and across regions, toward international steady states. Second, we evaluate whether convergence of CH$_4$ intensities drives convergence of CH$_4$ per capita. Third, we take different viewpoints on the responsibility for emissions and evaluate convergence of emissions from a production-based and from a consumption-based perspective. Finally, we take into account the importance of sectoral specialization for methane emissions and analyze convergence of emissions not only economy-wide but also for seven broad economic sectors.

The specification of our econometric model is motivated by the theoretical models of Brock and Taylor (2010) and Ordás Criado et al. (2011), which boil down to models of $\beta$-convergence.

Following Fernández-Amador et al. (2019), we implement a Bayesian structural model that addresses potential sources of endogeneity, accommodates heteroscedasticity in the data, and allows to test for group-specific convergence patterns. We test for the existence of group-specific convergence of CH$_4$ emissions for the European Union (EU), the Organisation of Economic Co-Operation and Development (OECD), and Annex I countries to the United Nations Framework Convention on Climate Change (UNFCCC) that ratified the Kyoto Protocol.

Our results indicate rapid convergence of all CH$_4$ inventories toward individual-specific steady states, suggesting that emission reductions will mainly be associated with long-run equilibrium growth paths. Convergence of CH$_4$ emissions toward global steady states is concentrated in specific economic sectors, and shows up on the economy-wide level only for CH$_4$ per capita embodied in consumption. Evidence for different group-specific convergence dynamics is weak and is present only for OECD and Annex I countries in very few economic sectors.

Moreover, our findings reveal differences in international convergence dynamics across emission inventories at the sectoral level. While production-based emission intensities of most sectors converge across countries, production-based emissions per capita do not, indicating that structural differences across countries may persist over time. Despite the lack of convergence in production-based CH$_4$ per capita across countries, international trade allows CH$_4$ per capita embodied in consumption to converge. Overall, our findings highlight that current patterns of methane emissions are unsustainable and that a large share of emission reductions will be related to the long-run equilibrium path, implying that mitigation policies could be costly. The sectoral differences in the convergence dynamics of methane inventories suggest that a differentiated, sectoral approach for multilateral agreements on methane emissions may be beneficial.

The paper is structured as follows. Section 2 outlines the empirical model and discusses the data. Section 3 summarizes the results for economy-wide and sectoral convergence of the four different methane inventories to individual-specific and global steady states. Section 4 provides a discussion of the findings and concludes.

## Econometric model and data

### The model

We test for $\beta$-convergence using a Bayesian structural model developed by Fernández-Amador et al. (2019), which is based on the Green Solow model (Brock and Taylor 2010) and the neoclassical growth model by Ordás Criado et al. (2011). In a nutshell, the test for $\beta$-convergence toward individual-specific steady states is implemented as

1 $$\begin{aligned} G_{i,t,s}= & {} \beta E_{i,t-s} + \sum _j{\left[ \beta _{j} d_j E_{i,t-s}\right] } + \pi _0 g_{i,t,s} + \pi _1 Y_{i,t-s} + \sum _r{\left[ \lambda _r z_{r,i,t-s}\right] } \nonumber \\&+ \delta _t + \alpha _i + \epsilon _{1,it} \end{aligned}$$

2 $$\begin{aligned}&\quad g_{i,t,s} = \alpha _{iv} + \beta _{iv} L(g_{i,t,s}) + \epsilon _{2,it} \end{aligned}$$

The dependent variable in Eq. (1) is the average growth rate of emissions in region *i* over the period $t-s$ and *t* ($G_{i,t,s}$), alternatively released from production and embodied in consumption, both per capita and per unit of value added. $G_{i,t,s}$ depends on the logarithm of lagged emissions ($E_{i,t-s}$), interactions of $E_{i,t-s}$ with dummy indicators for EU, OECD, and Annex I countries that ratified the Kyoto Protocol ($d_j$), the average growth rate and the lagged logarithm of real GDP per capita ($g_{i,t,s}$ and $Y_{i,t-s}$), a set of control variables ($z_{r,i,t-s}$), and time- and individual-specific effects ($\delta _t$ and $\alpha _i$). The control variables are motivated by previous literature (Richmond and Kaufmann 1997; Frankel and Rose 2005; Aichele and Felbermayr 2012, 2015; Fernández-Amador et al. 2017, 2019) and consist of the logarithm of population density, trade openness, a political regime index, the share of nuclear energy and fossil fuels in energy production, fossil rents as share of GDP, and value-added shares of seven sectors: livestock, energy, manufacturing, service, transport, and public administration, with the agricultural sector being the base category.

The average growth rates of emissions and GDP per capita are calculated as the difference of logarithms over $t-s$ and *t*, divided by the number of years, *s*. In our setting, *s* equals 3–4 years, which avoids potential upward bias in the estimated speed of convergence that could result from short-term fluctuations in emissions (Ordás Criado et al. 2011). Because the growth rate of GDP per capita may be jointly determined with the growth rate of emissions over the same period, we account for its potential endogeneity in Eq. (2). Following Barro and Sala-i-Martin (2004), we instrument the growth rate of GDP per capita with its growth rate in the previous period, $L(g_{i,t,s})$, where $L(\cdot )$ denotes the lag operator.

The parameter associated with lagged emissions, $\beta$, is of particular interest. Specifically, $\beta <0$ provides evidence for conditional $\beta$-convergence. Furthermore, the possibility of group-specific convergence is evaluated by including the interactions of lagged emissions with indicators for group membership in the EU, OECD, and Annex I ($d_j E_{i,t-s}$). The importance of group-specific convergence is tested using stochastic search variable selection (SSVS) priors (George and McCulloch 1993), which allow to derive posterior inclusion probabilities (PIPs) for the group-specific convergence terms. We conclude that there is evidence for a specific convergence pattern of group *j* under two conditions: $PIP_j >0.5$ and $\beta _j \ne 0$.

The system formed by Eqs. (1)–(2) is solved by a Cholesky rotation to represent the model in recursive form (see Lopes and Polson 2014; Fernández-Amador et al. 2019). Cross-sectional heteroscedasticity is operationalized through endogenously estimated individual-specific variances, modeled as a scale mixture of multivariate normal distributions; the estimate of the parameter $\nu$, which governs the distribution of the individual-specific weights, provides information about the degree of cross-sectional heteroscedasticity. All priors are specified as in Fernández-Amador et al. (2019), to which we refer for details.

Additionally to the specification explained above, we test for $\beta$-convergence toward international steady states conditional on socio-economic and political variables, by estimating the system formed by Eqs. (1)–(2) omitting the individual-specific effects, $\alpha _i$. Finally, we perform all the estimations using methane emissions at the sectoral level as the dependent variable.^4^

### The data

Global panel data on anthropogenic CH$_4$ emissions, disaggregated to 66 countries and 12 composite regions are available from Fernández-Amador et al. (2020b) for the years 1997, 2001, 2004, 2007, 2011, and 2014. The data cover production- and consumption-based CH$_4$ emissions. Production-based emissions correspond to territorial emissions, which constitute the basis for international agreements according to the polluter pays principle, while consumption-based emission inventories account for emissions released across the whole supply chain, allocating the responsibility for emissions to the consumer of final products. Apart from total releases of emissions, the database provides information on emissions per capita and emissions per unit of value added (emission intensities). Because emissions per capita are determined by emissions per unit of value added times value added per capita, the distinction between the two emission aggregates allows to evaluate whether changes in emissions per capita are driven by changes in emission intensities.

The data also provide information on emissions from 57 economic sectors, which are aggregated to seven broader economic sectors—namely agriculture, livestock, manufacturing, services, energy, transport, and public administration. Most of the 57 sectors do not release CH$_4$ or only account for a tiny share of CH$_4$ emissions produced. The main sources of anthropogenic CH$_4$ emissions are cattle farming and dairy (covered by the livestock sector); coal, oil, and gas production (energy sector); waste management (public administration sector); rice cultivation (agriculture sector); the transportation of fossil fuels (transport sector); and the petrochemical industry (manufacturing).^5^ Table 1 shows the average contribution of the seven broad sectors to aggregate CH$_4$ emissions between 1997 and 2014, for production and consumption-based emission inventories.

Global anthropogenic CH$_4$ emissions increased from about 8000 to nearly 9500 megatons of CO$_2$ equivalents, based on GWPs over 100 years (Fernández-Amador et al. 2020b). The sectoral distribution of emissions remained roughly constant over 1997–2014.

**Table 1:** Sectoral contributions to total CH$_4$ emissions

	Sectoral contribution in % (average 1997–2014)						
CH$_4$	Agriculture (%)	Livestock (%)	Energy (%)	Manufacturing (%)	Services (%)	Transport (%)	Public administration (%)
Production	8.68	35.05	24.00	5.04	0.50	5.56	21.16
Consumption	13.30	23.94	5.11	15.27	13.02	4.58	24.78

It is noteworthy that the bulk of emissions at the sectoral level is concentrated in few countries and regions. For example, the three largest polluters in the respective sectors account for more than half of worldwide CH$_4$ emissions from agriculture (China, India, and Indonesia) and services (the region Rest of Sub-Saharan Africa, China, and India); for about 45% of CH$_4$ from manufacturing (the region Rest of Middle East, Russia, and China) and from the energy sector (China, Rest of Middle East, and the USA); and for about a third of CH$_4$ from the livestock sector (India, Brazil, and Rest of Sub-Saharan Africa), the transport sector (Russia, USA, and Rest of Middle East), and the public administration sector (China, India, and the USA).

Data on the independent variables included in the regressions are sourced from various datasets. The World Bank’s World Development Indicators (WDI) provide data for GDP per capita in constant international dollars, population density, the shares of nuclear energy and fossil fuels in energy production, and fossil rents as share of GDP. Trade openness, measured as imports plus exports as a share of GDP, and value added shares of the agriculture, livestock, energy, manufacturing, service, transport, and public administration sectors are derived from the GTAP database. The political regime index, which ranges from $-10$ for strongly autocratic to $+10$ for strongly democratic regimes, is available from the Polity IV database. Table A.1 in the online Appendix provides details on the data sources, while descriptive statistics are provided in Table A.2.

## Results

### Convergence of economy-wide emissions

In this section, we analyze convergence of economy-wide emissions toward individual-specific steady states (Table 2, upper panel) and international convergence toward global steady states (Table 2, lower panel). Then, we turn to the sectoral results in Sect. 3.2.^6^

#### Individual-specific convergence

The tests for convergence of emissions toward individual-specific steady states (Table 2, upper panel) provide information on how far a country’s emissions are from their steady-state value, and, thus, on the degree to which emission reductions will be associated with transitional dynamics or with the long-run equilibrium growth path. This is informative about the costs of technology adoption for emission abatement. The results of the regressions provide evidence for conditional convergence of emissions within regions toward individual-specific steady states of all four CH$_4$ emission inventories. The estimates of the convergence parameter $\beta$ (i.e., of the logarithm of lagged emissions) are negative and different from zero at the 99% equal-tailed credible interval (CI). Like for CO$_2$ emissions (see Fernández-Amador et al. 2019), there is no evidence for faster group-specific convergence in EU, OECD, or Annex I countries—the interactions with the logarithm of lagged emissions are not different from zero at the 90% CI and the PIPs are very low.

**Table 2:** Convergence of economy-wide CH$_4$ emissions

	(1)	(2)	(3)	(4)
	CH$_4$ prod pc	CH$_4$ prod per VA	CH$_4$ cons pc	CH$_4$ cons per VA
**Individual-specific convergence**				
Ln(emissions)	−0.1214***	−0.1651***	−0.1735***	−0.0932***
Ln(emissions)$\cdot$EU	0.0054	0.0004	0.0000	0.0001
Ln(emissions)$\cdot$OECD	−0.0001	−0.0003	−0.0003	−0.0001
Ln(emissions)$\cdot$Annex I	0.0000	0.0000	0.0076	0.0003
Ln(income pc)	0.0273**	$-0.0604$***	0.0551***	−0.011
Income pc growth	0.0987	$-0.9573$***	0.7658***	$-0.5942$**
Additional controls	Yes	Yes	Yes	Yes
Time-dummies	Yes	Yes	Yes	Yes
Individual-dummies	Yes	Yes	Yes	Yes
PIP EU	0.1669	0.0079	0.0172	0.0159
PIP OECD	0.0219	0.0086	0.0168	0.0088
PIP Annex I	0.0038	0.0042	0.3359	0.0041
Half-life	5.4	3.8	3.6	7.1
$\nu$	3	4	4	4
DIC	$-3708$	$-3267$	$-3388$	$-3235$
*Instrumental equation*				
Constant	0.0145***	0.0145***	0.0146***	0.0146***
Income pc growth, lagged	0.3624***	0.3626***	0.3621***	0.3596***
**International convergence**				
Ln(emissions)	$-0.0014$	$-0.0038$	$-0.0176$***	$-0.0016$
Ln(emissions)$\cdot$EU	$-0.0001$	0.0006	0.0001	$-0.0002$
Ln(emissions)$\cdot$OECD	0.0002	$-0.0001$	0.0002	0.0001
Ln(emissions)$\cdot$Annex I	$-0.0002$	$-0.0001$	$-0.0005$	$-0.0004$
Ln(income pc)	$-0.0026$	0.0007	0.0083**	0.0008
Income pc growth	0.1603	$-0.7632$***	0.4399***	$-0.2538$
Additional controls	Yes	Yes	Yes	Yes
Time-dummies	Yes	Yes	Yes	Yes
Individual-dummies	No	No	No	No
PIP EU	0.0052	0.0019	0.0071	0.0017
PIP OECD	0.0031	0.0037	0.0068	0.0090
PIP Annex I	0.0035	0.0077	0.0248	0.0018
Half-life	494.8	182.1	39	432.9
$\nu$	3	4	4	4
DIC	$-3726$	$-3278$	$-3479$	$-3313$
*Instrumental equation*				
Constant	0.0145***	0.0145***	0.0145***	0.0146***
Income pc growth, lagged	0.3628***	0.3616***	0.3641***	0.3607***

*CI 90%, **CI 95%, ***CI 99% where CI stands for the equal-tailed credible interval. $N = 390$ observations. All variables but group dummies and income pc growth enter in lagged values. The half-life is calculated as $ln(0.5)/ln(1+\beta )$. $\nu$ measures the degree of cross-sectional heteroscedasticity; it governs the distribution of individual-specific weights of the scale mixture of normals, corresponding to a t-student distribution with $\nu$ degrees of freedom (see Fernández-Amador et al. 2019). The deviance information criterion (DIC) is computed as $DIC={\hat{D}}_q + Var(D_q)/2$, where $D_q$ is the deviance measure associated with draw *q* in the Markov Chains (see Spiegelhalter 2002; Gelman et al. 2004, Chap. 7). Results are based on 3 Markov Chains with 750,000 iterations each, after a burn-in of 750,000, from which every third draw is retained. More detailed results are reported in the online appendix (Tables A.3 and A.4)

The implied half-lives—i.e., the time needed to halve the distance between current and steady-state levels of emissions—range from 3.6 (consumption per capita) to 7.1 years (consumption per value added). Thus, CH$_4$ emissions per capita and CH$_4$ intensities are close to their country-specific steady states and emission reduction policies will mainly be related to the long-run equilibrium path of emissions, which implies potentially higher costs for emission abatement. These speeds of convergence are comparable to or slightly slower than those of CO$_2$ emissions (e.g., Westerlund and Basher 2008; Jobert et al. 2010; Fernández-Amador et al. 2019).

For production-based emission inventories, CH$_4$ intensities converge slightly faster than CH$_4$ per capita. Thus, convergence in emission intensities drives the convergence in emissions per capita. By contrast, for consumption-based inventories, CH$_4$ emissions per capita are very close to their steady state, but this result does not appear to be primarily driven by the dynamics in CH$_4$ intensities, which are further away from their steady-state levels. The slower convergence in CH$_4$ intensities of consumption may be driven by changes in the composition of consumption on the one hand, or by changes in the methane-intensity of methods to produce the consumption goods on the other hand—since the CH$_4$ intensity of production shows rather fast convergence, the reason for the slower convergence in CH$_4$ intensities of consumption is likely to be changes in consumption patterns.

Income per capita and its growth rate increase the growth rate of CH$_4$ emissions per capita and decrease the growth rate of CH$_4$ per value added. That is, income growth leads to a reduction in CH$_4$ intensity but also to an expansion of economic activity that counterbalances and outweighs this methane-efficiency gain, such that the growth rate of CH$_4$ per capita is larger at higher income levels. This is consistent with the predictions of the model by Ordás Criado et al. (2011) and may also explain the lack of empirical evidence for a negative slope in the income–CH$_4$ relationship, both over time (Jorgenson and Birkholz 2010) and at higher income levels (Fernández-Amador et al. 2018), which the environmental Kuznets’ curve hypothesis predicts.

#### International convergence across regions

Turning to international convergence across countries (Table 2, lower panel), the estimates for $\beta$ are not different from zero at the 90% CI but for consumption-based emissions per capita. Thus, only CH$_4$ per capita embodied in consumption converges across countries, while there is no evidence for international convergence of the other emission inventories. This contrasts with the findings of some empirical studies on CO$_2$ emissions, which find evidence for convergence (e.g., Nguyen 2005; Westerlund and Basher 2008; Brock and Taylor 2010; Fernández-Amador et al. 2019). Also, the implied half-life for CH$_4$ per capita embodied in consumption, the only inventory for which we found convergence, is with 39 years much longer than the 15 years found for CO$_2$ emissions by Fernández-Amador et al. (2019). Again, there is no evidence for faster group-specific convergence of CH$_4$ emissions, as the parameters associated with the interaction terms are not different from zero and their PIPs are very low. Interestingly, the effects of income per capita and its growth rate disappear for some emission inventories. The largely missing evidence for cross-country convergence of aggregate CH$_4$ emissions, especially for production-based emissions, questions the potential to achieve multilateral agreements for methane.^7^

The convergence of consumption-based emissions per capita is not associated with convergence of consumption-based emission intensities. In this sense, the convergence of emissions per capita is unlikely to be driven by converging consumption bundles across countries and may be related to the importation of products produced elsewhere as a result of income effects that impact on the scale of consumption. Furthermore, the absence of international convergence of CH$_4$ emissions released from production may result from persistent sectoral specialization patterns. If production structures only change very slowly, the concentration of CH$_4$ emissions in specific economic sectors may account for differences in CH$_4$ releases that persist over time. In the next subsection, we explore sector-specific convergence patterns in order to provide additional insights to interpret the economy-wide findings.

**Table 3:** Individual-specific convergence of sectoral CH$_4$ emissions

	Agriculture	Livestock	Energy	Manufacturing	Services	Transport	Public administration
*CH*$_4$ *Production per capita*							
Ln(emissions)	$-0.2230$***	$-0.2457$***	$-0.1524$***	$-0.2110$***	$-0.2140$***	$-0.1389$***	$-0.0670$***
Ln(emissions) EU	0.0014	0.0000	$-0.0009$	0.0001	0.0016	$-0.0013$	0.0005
Ln(emissions) OECD	$-0.0006$	$-0.0056$	$-0.0001$	0.0017	0.0003	$-0.0017$	0.0238
Ln(emissions) Annex I	0.0007	0.0000	$-0.0004$	0.0004	$-0.0007$	0.0001	0.0000
Ln(income pc)	$-0.0161$	0.0242***	0.0172	$-0.0246$	$-0.0448$	0.0096	0.0153
Income pc growth	0.4556	0.0182	0.4140	0.3333	0.2929	1.3243***	0.2264*
Half-life	2.7	2.5	4.2	2.9	2.9	4.6	10.0
DIC	- 2617	$$ - 3474$$	$$- 2791$$	$$-2646$$	$$- 2029$$	$$- 2687$$	$$- 3582$$
*CH*$_4$ *Production per value added*							
Ln(emissions)	$-0.2218$***	$-0.2102$***	$-0.2358$***	$-0.1925$***	$-0.2106$***	$-0.1739$***	$-0.1873$***
Ln(emissions) EU	$-0.0002$	$-0.0042$	0.0004	$-0.0002$	0.0015	0.0022	$-0.0004$
Ln(emissions) OECD	$-0.0005$	0.0002	0.0393	0.0003	$-0.0001$	-0.0005	0.0404
Ln(emissions) Annex I	-0.0223***	0.0019	-0.0001	0.0004	0.0002	$$-0.0007 $$	0.0006
Ln(income pc)	$-0.0529$*	$-0.0103$	0.0078	$-0.0801$**	$-0.1066$*	$-0.0601$*	$-0.0334$
Income pc growth	$$ -0.4692$$	$$-1.2311$$**	0.5570	$$-0.2868$$	$$-1.5272$$*	$$-0.1067 $$	$$-1.2120$$**
Half-life (Annex I)	2.8 (2.5)	2.9	2.6	3.2	2.9	3.6	3.3
DIC	$$- 2426$$	$$- 2620$$	$$- 2319$$	$$-2426$$	$$ - 1971$$	−2345	$$ - 2677$$
*CH*$_4$ *Consumption per capita*							
Ln(emissions)	$-0.2328$***	$-0.2314$***	$-0.2020$***	$-0.2044$***	$-0.1860$***	$-0.1866$***	$-0.1604$***
Ln(emissions) EU	0.0025	$$-0.0014 $$	$$ -0.0025$$	0.0030	0.0002	0.0007	0.0001
Ln(emissions) OECD	$$-0.0002 $$	0.0009	0.0009	$$-0.0002$$	$$-0.0015$$	$$-0.0014$$	0.0518*
Ln(emissions) Annex I	$$-0.0254$$**	0.0001	$$-0.0009$$	$$-0.0021$$	0.0001	$$ -0.0291$$**	0.0056
Ln(income pc)	0.0041	0.0098	$-0.0607$**	0.0419**	0.0380	0.0244	.0403***
Income pc growth	0.9372*	0.7786**	0.5062	1.3066***	2.2082***	1.9071***	0.7276***
Half-life (Annex I) [OECD]	2.6 (2.3)	2.6	3.1	3.0	3.4	3.4 (2.9)	4.0 [6.0]
DIC	$$- 2659$$	$$- 2858$$	$$-2597$$	$$- 2884$$	$$ - 2679$$	$$- 2504$$	$$- 3316 $$
*CH*$_4$ *Consumption per value added*							
Ln(emissions)	$-0.0705$***	$-0.0403$***	$-0.1124$***	$-0.0320$***	$-0.0129$***	$-0.0390$***	$-0.1070$***
Ln(emissions) EU	0.0012	0.0153	0.0030	$-0.0003$	0.0058	$-0.0003$	$-0.0004$
Ln(emissions) OECD	0.0001	$-0.0014$	0.0016	0.0028	0.0006	0.0019	$-0.0001$
Ln(emissions) Annex I	$-0.0072$	$-0.0011$	$-0.0015$	$-0.0015$	$-0.0003$	$-0.0003$	0.0007
Ln(income pc)	0.0123	0.0409	0.0515*	$$-0.0614$$***	0.0362	$$-0.0327 $$	$$-0.0243$$
Income pc growth	0.2971	$$-0.3109 $$	1.8663***	0.4292	$$-0.2917$$	0.3675	$$-0.2821$$
Half-life	9.5	16.9	5.8	21.3	53.4	17.4	6.1
DIC	$$ - 2481$$	$$- 2609$$	$$ - 2197 $$	$$- 2820$$	$$- 2591$$	−2700	$$-2901$$

*CI 90%, **CI 95%, ***CI 99%. Regressions include additional controls, time and individual dummies. Definitions as in Table 2.Results based on 3 Markov chains with 50,000 iterations each, after a burn-in of 25,000. For more detailed results, including IV, see Tables A.5–A.8 in the online appendix

### Convergence of sectoral emissions

#### Individual-specific convergence

The results from the sectoral convergence tests toward individual-specific steady states are summarized in Table 3. The upper two panels show the results for CH$_4$ per capita and per unit of value added released from production, and the lower two panels refer to emissions embodied in consumption.^8^

In line with the finding for aggregate emissions, there is strong evidence for convergence to individual-specific steady states for all methane inventories in all sectors, as shown by the posterior estimates of $\beta$, which are negative and different from zero at the 99% CI throughout. The speed of convergence and the implied half-lives at the sectoral level explain the findings at the economy-wide level. For most sectors and inventories, the half-lives range from 2.3 to 4.6 years. The exceptions are methane per capita from production in public administration, with a half-life of 10 years, and consumption-based methane intensities in all sectors, with half-lives up to 53 years in services. The relatively large contribution of public administration to economy-wide emissions (over 20%, see Table 1) explains the relatively slower speed of convergence found in CH$_4$ per capita from production economy-wide. By contrast, the relatively long half-life of consumption-based CH$_4$ intensity found economy-wide is explained by the slow convergence of consumption-based CH$_4$ intensity estimated in all sectors. Especially the long half-lives in the service, manufacturing, transport, and livestock sectors indicate that consumption patterns from these sectors are subject to change.

Notably, certain economic sectors are characterized by group-specific convergence of CH$_4$ emissions in Annex I countries on the one hand, and OECD countries on the other hand, which contributes further detail to the economy-wide findings. Yet, these group-specific convergence dynamics are only present in few economic sectors, and thus, they are too weak to show up in the economy-wide results summarized above. On the one side, Annex I countries show faster convergence of CH$_4$ emissions from agriculture (CH$_4$ intensity of production and CH$_4$ per capita consumption) and from the transport sector (CH$_4$ per capita consumption). Thus, the lower CH$_4$ intensity of agricultural production in Annex I countries observed in our data may be related to these differential (faster) convergence dynamics, whereas in non-Annex I countries the transition to lower CH$_4$ intensities is slower. Yet, the detected differential may also be related to heterogeneous sectoral specialization within the agriculture sector. In this sector, methane releases are mainly caused by rice cultivation, which accounts for only a small and decreasing share of agricultural activities in Annex I countries.

Turning to the consumption-based inventories, the speed of convergence of CH$_4$ intensity is similar across Annex I and non-Annex I countries in all sectors. Thus, the faster speed of convergence of CH$_4$ per capita in the agriculture and transport sectors in Annex I countries is likely to be driven by the scale of consumption, which appears to be closer to its steady-state level as compared to that of non-Annex I countries. On the other side, OECD countries exhibit slower convergence of consumption-based emissions per capita from public administration. The specific dynamics in OECD countries seem to be also related to the scale of consumption of goods and services from the public sector, since such a differential is not present when looking at consumption-based CH$_4$ intensity.

Similar to the economy-wide estimates, income per capita and its growth rate are connected to a higher growth rate of emissions per capita but to lower growth rate of methane intensities, whenever their effect is different from zero. The only exception is the energy sector, where for consumption-based emissions there is some evidence of the opposite effect. This suggests that in faster growing economies consumption from the energy sector may shift toward more oil and gas intensive products, such that CH$_4$ intensity increases. It also suggests that in periods of faster economic growth the income-elasticity of consumption from the energy sector is lower, which explains the lower growth rate of CH$_4$ per capita embodied in consumption. Overall, the income terms are different from zero more often for consumption-based than for production-based emissions per capita, whereas the opposite is true for methane intensities. This is in line with the slightly larger income-elasticity of economy-wide consumption-based relative to production-based per capita emissions found by Fernández-Amador et al. (2018) and the smaller income-elasticity of methane intensity of consumption generally found in sectoral estimations by Fernández-Amador et al. (2020a).

#### International convergence across regions

The results of the test for international convergence of sectoral methane emissions across regions are shown in Table 4. Although at the economy-wide level (in Sect. 3.1) there is no evidence for international convergence of most of the aggregate CH$_4$ inventories, the sectoral estimates suggest that emissions from many economic sectors converge toward global steady states, conditional on economic and political factors. More specifically, there is international convergence for all CH$_4$ inventories, with the exception of CH$_4$ per capita from production in livestock, energy, and transport, and the CH$_4$ intensity of consumption in agriculture, manufacturing, and services. The public administration sector only shows convergence toward global steady states for CH$_4$ per capita embodied in consumption. The speed of convergence (and divergence) varies across sectors but is slow in general, with half-lives ranging from 8 to 187 years.

**Table 4:** International convergence of sectoral CH$_4$ emissions

	Agriculture	Livestock	Energy	Manufacturing	Services	Transport	Public administration
*CH*$_4$ *production per capita*							
Ln(emissions)	–0.0037*	–0.0007	–0.0030	–0.0137***	–0.0287***	–0.0054	–0.0004
Ln(emissions) EU	−0.0017	–0.0014	0.0017	0.0018	0.0017	–0.0003	–0.0007
Ln(emissions) OECD	–0.0018	–0.0003	–0.0006	–0.0009	–0.0007	0.0013	0.0014
Ln(emissions) Annex I	–0.0025	0.0021	–0.0002	0.0010	–0.0012	0.0006	–0.0011
Ln(income pc)	–0.0004	0.0012	–0.0032	0.0086	–0.0070	0.0049	–0.0050
Income pc growth	1.0143***	–0.0960	0.6370**	–0.4111	0.4863	0.7984**	0.2726**
Half-life	187.0	–	–	50.2	23.8	–	–
DIC	–2694	$- 3552$	$- 2815$	$- 2644$	$-2082$	$-2665$	$-3562$
*CH*$_4$ *production per value added*							
Ln(emissions)	$$-0.0107$$***	$$-0.0211$$***	$$-0.0123$$***	$$-0.0227$$***	$$-0.0239$$***	$$-0.0152 $$***	$$-0.0039 $$
Ln(emissions) EU	$$-0.0004$$	$$-0.0008$$	0.0027	0.0020	$$- 0.0017 $$	0.0013	0.0001
Ln(emissions) OECD	$-0.0013$	0.0001	$-0.0004$	0.0002	$-0.0009$	0.0008	0.0220*
Ln(emissions) Annex I	0.0004	0.0002	0.0009	$-0.0008$	0.0002	0.0064	0.0003
Ln(income pc)	0.0147	0.0001	0.0004	$$-0.0076 $$	$$- 0.0458 $$**	$$- 0.0068$$	0.0006
Income pc growth	1.0057*	$$- 0.3009 $$	0.7413	$$- 1.4113$$**	$$- 0.4106$$	$$- 0.7394$$	$$- 0.0449$$
Half-life (OECD)	64.4	32.5	56.0	30.2	28.7	45.3	($-38.6$)
DIC	$- 2377$	$- 2585$	$- 2336$	$-2416$	$-2016$	$- 2341$	$- 2665$
*CH*$_4$ *consumption per capita*							
Ln(emissions)	$-0.0371$***	$-0.0181$***	$-0.0226$***	$-0.0562$***	$-0.0501$***	$-0.0821$***	$-0.0215$***
Ln(emissions) EU	$-0.0002$	$-0.0004$	0.0002	$-0.0004$	$-0.0009$	0.0018	0.0000
Ln(emissions) OECD	$-0.0011$	0.0002	0.0000	$-0.0017$	0.0003	0.0021	0.0002
Ln(emissions) Annex I	$-0.0012$	0.0001	$-0.0007$	0.0002	0.0001	$-0.0043$	$-0.0011$
Ln(income pc)	0.0164*	0.0009	0.0188*	0.0410***	0.0555***	0.0796***	0.0068
Income pc growth	1.1193**	$-0.2941$	0.9276*	0.4823	1.3760***	1.7038***	0.6050***
Half-life	18.3	37.9	30.3	12.0	13.5	8.1	31.9
DIC	$-2622$	$-2840$	$-2488$	$- 2914$	$- 2712$	$- 2546$	$-3383$
*CH*$_4$ *consumption per value added*							
Ln(emissions)	$-0.0032$	$-0.0164$***	$-0.0104$**	$-0.0012$	$-0.0021$	$-0.0115$***	$-0.0007$
Ln(emissions) EU	0.0026	$$- 0.0003 $$	$$ -0.0013 $$	0.0002	$$- 0.0025 $$	0.0008	$$- 0.0008 $$
Ln(emissions) OECD	0.0007	0.0008	$$-0.0002$$	$$- 0.0026$$	$$- 0.0017 $$	$$- 0.0015$$	0.0041
Ln(emissions) Annex I	0.0012	0.0004	$$- 0.0009 $$	0.0000	$$- 0.0009$$	$$ - 0.0013 $$	0.0002
Ln(income pc)	0.0129	0.0037	0.0165	$$-0.0135 $$**	$$ - 0.0077$$	$$- 0.0072$$	0.0006
Income pc growth	0.6236	$- 0.1654$	1.1656**	0.0095	$-0.0278$	0.3172	0.2812
Half-life	–	41.9	66.3	–	–	59.9	–
DIC	$$- 2644$$	$$-2749$$	$$- 2301 $$	$$- 3005$$	$$- 2781$$	$$ - 2844$$	$$- 2915$$

*CI 90%, **CI 95%, ***CI 99%. Regressions include additional controls, time and individual dummies. Definitions as in Table 2. Results based on 3 Markov chains with 50,000 iterations each, after a burn-in of 25,000. For more detailed results, including IV, see Tables A.9–A.12 in the online appendix

Since there is convergence of CH$_4$ per capita embodied in consumption in all economic sectors, this convergence shows up in the economy-wide results on aggregate emissions. For the remaining inventories, the sectors for which we detect international convergence account for 14% (CH$_4$ per capita from production), 34% (CH$_4$ intensity of consumption), and 79% (CH$_4$ intensity of production) of overall emissions, respectively (see Table 1). Although this share is high for the CH$_4$ intensity of production, the OECD group shows some (weak) evidence of divergence in the public administration sector, such that there is no evidence for global convergence of emissions economy-wide. Again, we do not find evidence for faster group-specific convergence for EU, OECD, or Annex I countries.

The CH$_4$ intensities of production may converge across countries in most sectors, because countries with larger potential for methane efficiency improvements are catching up with more methane-efficient countries. This is in line with the overall decrease in methane intensity observed in the period covered in our sample. Yet, the estimated half-lives are relatively long, emphasizing a slow catch-up process. Nevertheless, as can be concluded from the economy-wide results on aggregate emissions, higher demand induced by global income expansion increases production-based emissions per capita, which seems to work against convergence forces. This scale effect is sizable and counteracts the convergence dynamics of CH$_4$ intensities in sectors comprising methane-intensive activities, such as cattle breeding in the livestock sector, drilling and transportation of fossil fuels in the energy and transport sectors, respectively, and waste management in the public administration sector (see Fernández-Amador et al. 2020b). Even though rice cultivation is CH$_4$ intensive, it only accounts for about 6% of value added from the agriculture sector, what may explain that there is some evidence for convergence of CH$_4$ per capita from agricultural production (90% CI). By contrast, the activities in the manufacturing sector account for only a small share of aggregate CH$_4$ releases (see Table 1). There, the scale effect of increased production does not outweigh the convergence forces of CH$_4$ intensities. Moreover, in the services sector, which accounts for merely 0.5% of aggregate emissions, the scale effect seems to strengthen convergence forces, such that the half-life is shorter for emissions per capita than for emission intensity.

Turning to consumption-based emissions, the convergence of CH$_4$ embodied in consumption per capita, detected for all sectors, is not driven by convergence in CH$_4$ intensities of consumption. CH$_4$ intensities of consumption are largely determined by the composition of consumption bundles and tastes. Consumer tastes and consumption patterns are usually rigid and to a large degree region specific (York and Gossard 2004; World Bank Group 2015), which may explain the absence of international convergence of CH$_4$ intensities of consumption detected in many sectors. Our results suggest, however, that there may be some assimilation in consumer tastes across countries in terms of demand for meat (livestock sector; see also York and Gossard 2004; Fiala 2008) and energy (energy and transport sectors), which shows up in converging CH$_4$ intensities of consumption from these sectors.

Finally, the income terms are estimated to be different from zero in about half of the regressions. In these cases, and as expected, increasing income is connected to a higher growth rate of emissions per capita and to a lower growth rate of CH$_4$ intensities. One exception is again the energy sector, and there is slight evidence that the CH$_4$ intensity of agricultural production increases faster (or decreases slower) if GDP per capita grows faster (90% CI).^9^

## Conclusion

In this study, we conducted a comprehensive analysis of convergence patterns of CH$_4$ emissions based on international data on anthropogenic CH$_4$ emissions, which contributes several insights to the literature on the sustainability of GHG emissions and provides information for international policy dialogue.

First, our results show that methane emissions per capita and CH$_4$ intensities are stabilizing rapidly around individual-specific steady states. The estimated short half-lives of CH$_4$ emissions (3.6–7.1 years) suggest that the steady-state level of emissions will not differ substantially from current levels. Since countries are already close to their steady-state levels of emissions, it is unlikely that methane releases will decrease substantially in the absence of policies specifically aimed at improvements in methane efficiency.

Although we find economic growth and development to be related to methane-efficiency gains—i.e., reductions of emissions per unit of value added—these gains do not counterbalance the effects of income expansion, which are reflected in rising CH$_4$ emissions per capita. This underlines the importance of policies to actively reduce CH$_4$ emissions, which could limit global warming in the near term (Jackson 2009; Shindell et al. 2012, 2017). Such policies may aim at improved water management in rice cultivation, nutritional changes of ruminants, adaptation of manure- and waste management systems, programs to reduce meat consumption, increased efforts to recover methane in coal production, reducing methane leakage by intensified inspections in oil and gas production and transportation, or the promotion of renewable energy sources (see Michaelowa and Dransfeld 2008; Fumoto et al. 2010; Höglund-Isaksson 2012; Gerber et al. 2013; Cowley and Brorsen 2018).

Second, our results do not provide evidence for international convergence of CH$_4$ emissions. This is a consequence of the concentration of CH$_4$ emissions in specific economic sectors and persistent patterns of economic specialization. Countries specialized in methane-intensive economic sectors may face difficulties to reduce their methane emissions, especially if demand for these sectors is increasing. This may explain why Argentina, Brazil, and Uruguay, all having large livestock sectors, have been particularly concerned with the specific treatment of CH$_4$ emissions in international cooperation mechanisms under Article 6 of the Paris agreement (see Carbon Brief 2019). Furthermore, despite persistent production structures, CH$_4$ emissions embodied in consumption per capita converge across countries, as a result of international trade. This difference in emission dynamics between production- and consumption-based per capita emissions highlights the relevance of the discussion about whether producers or consumers should be held responsible for emissions.

Third, at the sectoral level, we document important differences in international convergence dynamics between CH$_4$ emissions per capita and CH$_4$ intensities and between production- and consumption-based emissions. For production-based emissions, CH$_4$ intensities converge across countries in most economic sectors, suggesting that CH$_4$-intensive countries are catching up in terms of the CH$_4$ efficiency of production. Yet, international convergence in CH$_4$ per capita released from production is absent in methane-intensive sectors such as livestock, energy, transport, and public administration. This absence of convergence stems from the rather rigid nature of economic specialization patterns and the concentration of methane-intensive industries in few countries. In this context, rising global demand, which is met by expanding production, will especially increase emissions in countries that are specialized in methane-intensive industries. In this sense, national policies that target territorial emissions, such as the policies defined within the framework of NDCs, may not be effective in limiting territorial emissions per capita, which will be affected by expansions in global demand; national regulation could be more effective in lowering the emission intensity of production. Policies aimed at production-based emission intensities could be supplemented by policies that target consumption-based emissions per capita. In this vein, our result that consumption-based emissions per capita converge across countries suggests that policies targeted at consumption-based emissions may generate less concerns from an equity perspective and facilitate negotiations about a global climate policy framework.

International negotiations on the implementation of global markets for GHGs have not been successful so far. Negotiations at the COP25 in Madrid on the set-up of a global emission trading system have failed, in part because negotiators did not agree on the specific treatment of CH$_4$ emissions in aggregate GHG budgets (Carbon Brief 2019). Given the specificity of methane and the remarkable differences it shows with respect to CO$_2$, it seems appropriate to think about its separate treatment. In this regard, there is a trade-off between economic efficiency, which calls for regulation of aggregate GHG baskets, and voluntary participation in and compliance with international agreements, which could be better achieved by a separate and sector-specific treatment of CH$_4$ emissions (see Barrett 2008b). On the one side, the main economic argument in favor of aggregating different GHGs together and making baskets of CO$_2$ equivalents subject to emission trading is that this approach allows emissions to be reduced where the marginal costs of abatement are the lowest. On the other side, treating different GHGs separately may increase the probability to reach an agreement on the implementation of global markets for emissions. A separate agreement for CH$_4$ emissions could promote the voluntary participation of countries by reducing uncertainty connected to the conversion of CH$_4$ emissions to CO$_2$ equivalents. At the same time, a differentiated treatment of GHGs may allow policy makers to adapt climate policies more closely to the specific nature of different GHGs (see, e.g., Jackson 2009; Shindell et al. 2017; Fernández-Amador et al. 2020b).

Furthermore, the differences in the processes that generate methane emissions, which are characterized by different abatement potentials, and the specialization-related differences in emission dynamics across countries suggest that a strategy based on sector-specific regulation could be justified. Given the current deadlock of negotiations on the implementation of global carbon markets to operationalize the Paris Agreement, the possibility of designing specific agreements for CH$_4$ should be considered. Moreover, the bulk of emissions at the sectoral level is concentrated in few countries, thus reducing the number of negotiators with strong policy preferences. Therefore, a sector-specific treatment of CH$_4$ emissions may facilitate reaching consensus on targets, voluntary participation in the agreement, and compliance with its content, what is particularly relevant because of the horizontal nature of international law (see Barrett 2008a, b).

## Supplementary Information

Below is the link to the electronic supplementary material.

Supplementary material 1 (pdf 274 KB)

## Footnotes

1 For studies that find evidence for convergence or convergence clubs, see (Strazicich and List 2003);(Nguyen 2005);(Aldy 2006);(Barassi et al. 2011);(Romero-Ávila 2008);(Westerlund and Basher 2008);(Panopoulou and Pantelidis 2009);(Brock and Taylor 2010);(Jobert et al. 2010);(Ordás Criado and Grether 2011);(Camarero et al. 2013);(Yavuz and Yilanci 2013) and Fernández-Amador et al. (2019). For studies that do not find evidence for convergence, see Barassi et al. (2008) and Nourry (2009).

2 Production-based emissions convergence has been evaluated using tests for $\beta$, $\sigma$, and stochastic convergence. For $\beta$-convergence of CO$_2$ emissions, see, e.g., Strazicich and List (2003), Nguyen (2005), Brock and Taylor (2010), Jobert et al. (2010), Fernández-Amador et al. (2019), Karakaya et al. (2019), for $\sigma$-convergence see Aldy (2006), Panopoulou and Pantelidis (2009), Brock and Taylor (2010), Ordás Criado and Grether (2011), Camarero et al. (2013) and for stochastic convergence Strazicich and List (2003), Nguyen (2005), Aldy (2006), Romero-Ávila (2008), Barassi et al. (2008), Westerlund and Basher (2008), Nourry (2009), Barassi et al. (2011), Yavuz and Yilanci (2013). Tests for $\sigma$ and stochastic convergence are not applicable when testing for convergence of consumption-based emissions, since data availability is restricted in time by the availability of input–output (IO) tables needed to calculate consumption-based emissions. Also, for global samples of countries GHG emissions are likely to be affected by transition dynamics, which may pose issues for tests of stochastic convergence (Bernard and Durlauf 1996).

3 See, e.g., Peters (2008), Peters and Hertwich (2008), Wiedmann (2009), Wiedmann et al. (2011), Wiedmann et al. (2015), Davis and Caldeira (2010), Lenzen et al. (2012), Blanco et al. (2014), and Fernández-Amador et al. (2016).

4 We also implement a sensitivity analysis on the degree of heteroscedasticity of the model for economy-wide emissions. Two experiments are carried out. First, we specify alternative values of the hyperprior of the hyperparameter $\nu$. Second, we impose different values for $\nu$ exogenously. These experiments highlight the robustness of our results. See Appendix A.4.

5 See Fernández-Amador et al. (2020b) for further details on the contributions of the 57 sectors to total methane emissions.

6 Detailed regression results including the coefficients of the control variables and the instrumental equation, and robustness checks are reported in the online Appendix.

7 The very low estimates for $\nu$ throughout all regressions, which are robust to specifying alternative priors for $\nu$ (see Sect. A.4 in the online appendix), indicate the presence of heteroscedasticity. A sensitivity analysis for different exogenously set values of $\nu$ emphasizes that the main results are robust across a wide range of values (Table A.13 in the online appendix). Only the convergence term of CH$_4$ per value added of production becomes different from zero at the 90% CI for values for $\nu$ of 20 or higher, indicating very slow convergence toward global steady states.

8 More detailed results are available in Tables A.5–A.8 in the online appendix.

9 The evidence shown for agriculture seems to be driven by the fast growth rate of China, a large rice producer, since the international convergence regressions do not account for individual-specific effects. In the period covered in the data, the CH$_4$ intensity of agricultural production in China decreased more slowly in periods of higher economic growth.
