# Supplementary material for: Do methane emissions converge? Evidence from global panel data on production- and consumption-based emissions
Source: Empir Econ. 2021 Dec 9;63(2):877–900. doi: 10.1007/s00181-021-02162-9 (PMC9307563; doi:10.1007/s00181-021-02162-9)
Supplement: Supplementary file 1 — Supplementary material 1 (pdf 274 KB) [file 181_2021_2162_MOESM1_ESM.pdf]

A Online Appendix

A.1 Data and descriptive statistics

Variable	Description	Source
	Dependent variables	
Growth of CH_4 pc prod.	First difference of the natural logarithm of production-based CO_2 emissions per capita divided by the length of the period.	Fernández-Amador et al. (2020b)
Growth of CH_4 pc cons.	First difference of the natural logarithm of consumption-based CO_2 emissions per capita divided by the length of the period.	Fernández-Amador et al. (2020b)
Growth of CH_4 va prod.	First difference of the natural logarithm of production-based CO_2 emissions per value added divided by the length of the period.	Fernández-Amador et al. (2020b)
Growth of CH_4 va cons.	First difference of the natural logarithm of consumption-based CO_2 emissions per value added divided by the length of the period.	Fernández-Amador et al. (2020b)
	Control variables	
Ln(CH_4 pc prod.)	Natural logarithm of production-based CH_4 emissions per capita.	Fernández-Amador et al. (2020b)
Ln(CH_4 pc cons.)	Natural logarithm of consumption-based CO_2 emissions per capita.	Fernández-Amador et al. (2020b)
Ln(CH_4 va prod.)	Natural logarithm of production-based CH_4 emissions per value added.	Fernández-Amador et al. (2020b)
Ln(CH_4 va cons.)	Natural logarithm of consumption-based CH_4 emissions per value added.	Fernández-Amador et al. (2020b)
EU	Dummy = 1 for members of the European Union.	EU
OECD	Dummy = 1 for OECD members.	OECD
Annex I	Dummy = 1 for members of Annex I of the UNFCCC that ratified the Kyoto protocol.	United Nations
Ln(Income pc)	Natural logarithm of real GDP (PPP) per capita.	WDI
Income pc growth	First difference of the natural logarithm of real GDP (PPP) per capita divided by the length of the period (computed over the same period as the dependent variable).	WDI
Income pc growth, lagged	Income pc growth lagged by one period.	WDI
Ln(pop. density)	Log of number of inhabitants per square kilometer.	WDI
Openness	Trade openness calculated as (Exports+Imports)/GDP.	GTAP
Political regime	Polity2 political regime index.	Polity IV
Nuclear %	Share of nuclear energy in total energy production[a].	WDI
Fossil %	Share of fossil fuels in total energy production[a].	WDI
Fossil rents	Rents from fossil fuel production as share of GDP[a].	WDI
VA agriculture	Share of VA in agriculture relative to total VA.	GTAP
VA cattle	Share of VA in livestock relative to total VA.	GTAP
VA energy	Share of VA in energy sectors to total VA.	GTAP
VA manufacturing	Share of VA in manufacturing sectors relative to total VA.	GTAP
VA services	Share of VA in service sectors relative to total VA.	GTAP
VA transport	Share of VA in transport sectors relative to total VA.	GTAP
VA public admin.	Share of VA in public administration relative to total VA.	GTAP

[a] Values for composite regions were obtained as GDP weighted averages. If data was missing for individual group members, group averages were used.

Table A.1: Definition of variables and data sources

Table A.2: Descriptive statistics

	N	Mean	Std. Dev	Min	Max
		Dependent variables			
Growth CH_4 pc prod.	390	- 0.0046	0.0350	- 0.2938	0.3022
Growth CH_4 pc cons.	390	- 0.0018	0.0395	- 0.2792	0.2271
Growth CH_4 va prod.	390	- 0.0343	0.0492	- 0.3330	0.2057
Growth CH_4 va cons.	390	- 0.0318	0.0464	- 0.2720	0.2052
		Control variables			
Ln(CH_4 pc prod.)	390	0.0483	0.6840	- 1.6289	2.0115
Ln(CH_4 pc cons.)	390	0.2448	0.5808	- 1.3806	1.6543
Ln(CH_4 va prod.)	390	- 1.3398	1.4287	- 4.9462	1.4081
Ln(CH_4 va cons.)	390	- 1.3398	1.4287	- 4.9462	1.4081
EU	390	0.3128	0.4642	0	1
OECD	390	0.3872	0.4877	0	1
Annex I	390	0.3718	0.4839	0	1
Ln(income pc)	390	9.4981	1.1013	6.2054	11.4913
Income pc growth	390	0.0260	0.0267	- 0.1240	0.1194
Income pc growth, lagged	390	0.0278	0.0273	- 0.1240	0.1194
Ln(pop. density)	390	4.3048	1.4577	0.8798	8.9042
Openness	390	0.8212	0.4753	0.1761	3.2739
Political regime	390	6.2256	5.1222	- 7	10
Nuclear %	390	0.0984	0.1789	0	0.8357
Fossil %	390	0.5807	0.3060	0.0008	1
Fossil rents	390	0.0275	0.0584	0	0.4056
VA agriculture	390	0.0963	0.0882	0.0054	0.5063
VA cattle	390	0.0296	0.0211	0.0018	0.1195
VA energy	390	0.0712	0.0756	0.0014	0.4313
VA manufacturing	390	0.1653	0.0725	0.0238	0.4870
VA services	390	0.4409	0.1006	0.1414	0.6722
VA transport	390	0.0597	0.0336	0.0001	0.2614
VA public admin.	390	0.1370	0.0489	0.0003	0.2641

A.2 Detailed results for economy-wide emissions

Table A.3: Individual-specific convergence of economy-wide emissions

	(1) CH_4 pc prod.		(2) CH_4 pc cons.		(3) CH_4 VA prod.		(4) CH_4 VA cons.	
Outcome equation								
Constant	- 0.1699		- 0.3288	**	0.4362	***	0.2293	*
Ln(emissions)	- 0.1214	***	- 0.1735	***	- 0.1651	***	- 0.0932	***
Ln(emissions)·EU	0.0054		0.0000		0.0004		0.0001	
Ln(emissions)·OECD	- 0.0001		- 0.0003		- 0.0003		- 0.0001	
Ln(emissions)·Annex I	0.0000		0.0076		0.0000		0.0003	
Ln(income pc)	0.0273	**	0.0551	***	- 0.0604	***	- 0.0110	
Income pc growth	0.0987		0.7658	***	- 0.9573	***	- 0.5942	**
Ln(pop. density)	- 0.0345	*	- 0.0627	***	- 0.0274		- 0.0569	*
Openness	0.0000		0.0001		- 0.0003	**	- 0.0002	
Political Regime	0.0003		- 0.0001		- 0.0003		- 0.0013	
Nuclear %	0.0002		0.0006	**	- 0.0001		0.0000	
Fossil fuels %	- 0.0001		0.0004	*	- 0.0003		0.0000	
Fossil rents	0.0019	**	0.0017		0.0019		0.0016	
VA cattle	0.0015		0.0018		0.0037	*	0.0006	
VA energy	0.0005		0.0008		0.0003		- 0.0022	**
VA manufacturing	0.0008	*	0.0008		0.0019	**	0.0010	
VA services	0.0004		0.0007		0.0007		0.0004	
VA transport	0.0010		0.0008		0.0004		- 0.0001	
VA public admin.	0.0008	*	0.0001		0.0004		0.0000	
2004	0.0017		0.0074		- 0.0224	***	- 0.0143	***
2007	- 0.0053		- 0.0090		- 0.0281	***	- 0.0268	***
2011	- 0.0045		- 0.0149	**	- 0.0306	***	- 0.0310	***
2014	- 0.0081		- 0.0150	**	- 0.0423	***	- 0.0300	***
Individual-dummies	yes		yes		yes		yes	
Instrumental equation for income pc growth								
Constant	0.0145	***	0.0146	***	0.0145	***	0.0146	***
Income pc growth, lagged	0.3624	***	0.3621	***	0.3626	***	0.3596	***
a_{iv}	0.1195		-0.3224	*	0.2844		-0.1899	
PIP EU	0.1669		0.0172		0.0079		0.0159	
PIP OECD	0.0219		0.0168		0.0086		0.0088	
PIP Annex B	0.0038		0.3359		0.0042		0.0041	
Half-life	5.4		3.6		3.8		7.1	
ν	3		4		4		4	
DIC	- 3708		- 3388		- 3267		- 3235	
N	390		390		390		390	

Note: * CI 90%, ** CI 95%, *** CI 99% where CI stands for the equal-tailed credible interval. All variables but group dummies and income pc growth enter in lagged values. a_{iv} measures the strength of the error correlations of the instrumental and the outcome equation. PIP stands for the posterior inclusion probability of group-specific convergence dynamics. The half-life is calculated as $ln(0.5)/ln(1+\beta)$. ν measures the degree of cross-sectional heteroscedasticity; it governs the distribution of individual-specific weights of the scale-mixture of normals, corresponding to a t-student distribution with ν degrees of freedom. The Deviance Information Criterion (DIC) is computed as $DIC = \hat{D}_q + Var(D_q)/2$, where D_q is the deviance measure associated with draw q in the MC (see Spiegelhalter, 2002, Gelman et al., 2004, Chap. 7). Results are based on 3 MC with 750,000 iterations each, after a burn-in of 750,000, from which every third draw is retained. See Fernández-Amador et al. (2019) for further details on the model parameters.

Table A.4: International convergence of economy-wide emissions

	(1) CH_4 pc prod.		(2) CH_4 pc cons.		(3) CH_4 VA prod.		(4) CH_4 VA cons.	
Outcome equation								
Constant	0.0148		- 0.0565	**	0.0420		0.0351	
Ln(emissions)	- 0.0014		- 0.0176	***	- 0.0038		- 0.0016	
Ln(emissions)·EU	- 0.0001		0.0001		0.0006		- 0.0002	
Ln(emissions)·OECD	0.0002		0.0002		- 0.0001		0.0001	
Ln(emissions)·Annex I	- 0.0002		- 0.0005		- 0.0001		- 0.0004	
Ln(income pc)	- 0.0026		0.0083	**	0.0007		0.0008	
Income pc growth	0.1603		0.4399	***	- 0.7632	***	- 0.2538	
Ln(pop. density)	- 0.0018		- 0.0036	**	- 0.0043	**	- 0.0048	**
Openness	0.0000		0.0001	*	0.0000		0.0000	
Political Regime	- 0.0004	*	- 0.0004		- 0.0006		- 0.0009	**
Nuclear %	0.0000		0.0000		0.0001		0.0001	
Fossil fuels %	0.0000		- 0.0001		0.0000		0.0000	
Fossil rents	0.0003		0.0004		0.0010		0.0014	**
VA cattle	0.0001		- 0.0007		- 0.0005		- 0.0021	*
VA energy	- 0.0004		- 0.0006		- 0.0018	***	- 0.0023	***
VA manufacturing	0.0001		- 0.0002		0.0000		- 0.0001	
VA services	0.0000		- 0.0004		- 0.0006		- 0.0005	
VA transport	0.0003		0.0004		0.0001		0.0005	
VA public admin.	0.0001		0.0000		- 0.0010	**	- 0.0004	
2004	0.0091	***	0.0181	***	- 0.0049		- 0.0051	
2007	0.0067	**	0.0059		0.0110	**	- 0.0028	
2011	0.0091	***	0.0043		0.0157	***	- 0.0050	
2014	0.0097	***	0.0052		0.0150	***	0.0066	
Individual-dummies	no		no		no		no	
Instrumental equation for income pc growth								
Constant	0.0145	***	0.0145	***	0.0145	***	0.0146	***
Income pc growth, lagged	0.3628	***	0.3641	***	0.3616	***	0.3607	***
a_{iv}	0.0350		- 0.0710		- 0.0445		- 0.6900	***
PIP EU	0.0052		0.0071		0.0019		0.0017	
PIP OECD	0.0031		0.0068		0.0037		0.0090	
PIP Annex I	0.0035		0.0248		0.0077		0.0018	
Half-life	494.8		39.0		182.1		432.9	
ν	3		4		4		4	
DIC	-3726		-3479		-3278		-3313	
N	390		390		390		390	

Note: * CI 90%, ** CI 95%, *** CI 99%. Further information is provided in the notes to Table A.3.

A.3 Detailed results for sectoral emissions

A.3.1 Individual-specific convergence

Table A.5: Detailed results for individual-specific convergence of CH_4 production per capita

	agriculture	livestock	energy	manufacturing	services	transport	public admin.
**Individual-specific convergence: CH_4 production per capita**							
Constant	-0.1113	-0.1588	-0.1457	0.0227	-0.0192	-0.1191	-0.2043 **
Ln(emissions)	-0.2230 ***	-0.2457 ***	-0.1524 ***	-0.2110 ***	-0.2140 ***	-0.1389 ***	-0.0670 ***
Ln(emissions) EU	0.0014	0.0000	-0.0009	0.0001	0.0016	-0.0013	0.0005
Ln(emissions) OECD	-0.0006	-0.0056	-0.0001	0.0017	0.0003	-0.0017	0.0238
Ln(emissions) Annex I	0.0007	0.0000	-0.0004	0.0004	-0.0007	0.0001	0.0000
Ln(income pc)	-0.0161	0.0242 ***	0.0172	-0.0246	-0.0448	0.0096	0.0153
Income pc growth	0.4556	0.0182	0.4140	0.3333	0.2929	1.3243 ***	0.2264 *
Ln(pop. density)	-0.1823 ***	-0.0978 ***	-0.0980 ***	-0.1715 ***	-0.3188 ***	-0.1320 ***	-0.0098
Openness	0.0001	-0.0001	-0.0002	0.0000	0.0013 **	-0.0001	0.0000
Political regime	0.0000	0.0004	0.0006	-0.0020	0.0009	0.0013	0.0001
Nuclear %	-0.0015	0.0006 **	0.0001	-0.0004	0.0008	0.0005	0.0001
Fossil fuels %	-0.0004	0.0002	0.0002	-0.0005	0.0008	0.0000	-0.0001
Fossil rents	-0.0034	0.0017 *	0.0024	0.0069 **	0.0084 **	0.0054 **	0.0008
VA cattle	-0.0009	0.0027	-0.0029	0.0040	-0.0058	0.0031	0.0013
VA energy	0.0007	0.0004	-0.0011	0.0010	0.0023	0.0007	0.0003
VA manufacturing	0.0012	0.0010	0.0006	0.0016	0.0034	-0.0009	0.0004
VA services	0.0019	0.0002	-0.0005	0.0010	0.0029	0.0002	0.0000
VA transport	0.0004	0.0021 **	-0.0001	0.0032	0.0029	0.0060 **	0.0005
VA public admin.	0.0003	0.0013 **	0.0001	0.0042 **	0.0011	0.0000	-0.0001
2004	0.0214 **	-0.0082 **	0.0235 ***	0.0157	0.0217	-0.0069	0.0005
2007	0.0147	-0.0131 ***	0.0186 *	0.0079	0.0178	-0.0157	-0.0036
2011	0.0655 ***	-0.0156 ***	0.0125	-0.0490 ***	0.1266 ***	0.0135	-0.0007
2014	0.0562 ***	-0.0228 ***	0.0204 *	-0.0085	0.1000 ***	0.0156	-0.0036
Individual-dummies	yes	yes	yes	yes	yes	yes	yes
R^2	0.5610	0.9418	0.4603	0.4866	0.4904	0.4774	0.4020
DIC	-2617	-3474	-2791	-2646	-2029	-2687	-3582
Instrumental equation for income pc growth							
Constant	0.0144 ***	0.0145 ***	0.0144 ***	0.0145 ***	0.0144 ***	0.0143 ***	0.0144 ***
Income pc gr, lagged	0.3645 ***	0.3619 ***	0.3642 ***	0.3642 ***	0.3644 ***	0.3670 ***	0.3632 ***
a_{iv}	-0.6935	0.2048	-0.1130	0.1628	-0.3299	-0.8170 *	-0.1197
R^2	0.5312	0.5306	0.5300	0.5313	0.5298	0.5306	0.5301
PIP EU	0.0055	0.0327	0.0635	0.0076	0.0079	0.0055	0.0173
PIP OECD	0.0151	0.1188	0.0123	0.1063	0.0189	0.0109	0.6039
PIP Annex I	0.0065	0.0133	0.0109	0.0634	0.0051	0.0053	0.0150
Half-life	2.7	2.5	4.2	2.9	2.9	4.6	10.0
ν	3	3	3	4	3	3	3
N	390	390	390	390	390	390	390

Note: * CI 90%, ** CI 95%, *** CI 99%. a_{iv} is the strength of the correlation between the errors of the instrumental and the outcome equation. All variables but group dummies and income pc growth enter in lagged values. The half-life is calculated as $ln(0.5)/ln(1+\beta)$. The Bayesian R^2 is the mean of the R^2 computed for each draw q of the Markov chain (MC), R_q^2, where $R_q^2 = \sum_{i,t} \hat{y}_{it} / \left(\sum_{i,t} \hat{y}_{it} + \sum_{i,t} \epsilon_{it} \right)$, where $\hat{y}_{it}$ is the estimate of y_{it} implied by the model and $\epsilon_{it} = y_{it} - \hat{y}_{it}$ (see Gelman et al., 2017). The Deviance Information Criterion (DIC) is computed as $DIC = \hat{D}_q + Var(D_q)/2$, where D_q is the deviance measure associated with draw q in the MC (see Spiegelhalter, 2002, Gelman et al., 2004, Chap. 7). Results are based on 3 MC with 50000 iterations each, after a burn-in of 25000.

Table A.6: Detailed results for individual-specific convergence of CH_4 consumption per capita

	agriculture	livestock	energy	manufacturing	services	transport	public admin.
**Individual-specific convergence: CH_4 consumption per capita**							
Constant	-0.1655	-0.1954	-0.1810	-0.2621 **	-0.2507 *	-0.1860	-0.3213 ***
Ln(emissions)	-0.2328 ***	-0.2314 ***	-0.2020 ***	-0.2044 ***	-0.1860 ***	-0.1866 ***	-0.1604 ***
Ln(emissions) EU	0.0025	-0.0014	-0.0025	0.0030	0.0002	0.0007	0.0001
Ln(emissions) OECD	-0.0002	0.0009	0.0009	-0.0002	-0.0015	-0.0014	0.0518 *
Ln(emissions) Annex I	-0.0254 **	0.0001	-0.0009	-0.0021	0.0001	-0.0291 **	0.0056
Ln(income pc)	0.0041	0.0098	-0.0607 **	0.0419 **	0.0380	0.0244	0.0403 ***
Income pc growth	0.9372 *	0.7786 **	0.5062	1.3066 ***	2.2082 ***	1.9071 ***	0.7276 ***
Ln(pop. density)	-0.1078 ***	-0.0801 ***	0.0236	-0.1701 ***	-0.0991 ***	-0.1748 ***	-0.0743 ***
Openness	-0.0002	0.0002	0.0003	0.0000	0.0000	-0.0001	0.0001
Political regime	-0.0004	0.0007	-0.0008	-0.0019	-0.0013	-0.0013	0.0009
Nuclear %	0.0009	0.0008	-0.0001	0.0009	0.0012	0.0022 **	0.0008 **
Fossil fuels %	0.0015 **	0.0005	0.0018 ***	0.0005	0.0008	0.0018 **	0.0005 *
Fossil rents	-0.0004	0.0006	0.0028	0.0021	0.0028	0.0023	0.0014
VA cattle	-0.0007	0.0005	-0.0054	-0.0004	-0.0023	-0.0028	0.0015
VA energy	0.0011	0.0011	-0.0025	0.0028 *	-0.0018	0.0004	0.0006
VA manufacturing	-0.0011	0.0025	-0.0017	0.0013	-0.0014	-0.0005	0.0003
VA services	-0.0008	0.0007	-0.0020	0.0021 *	-0.0024	0.0000	0.0003
VA transport	0.0003	-0.0007	-0.0041 *	0.0023	-0.0028	0.0003	0.0008
VA public admin.	-0.0008	0.0008	0.0025	0.0004	-0.0024	-0.0029	-0.0009
2004	-0.0078	-0.0200 ***	0.0058	0.0320 ***	0.0760 ***	0.0319 **	0.0001
2007	-0.0270 *	-0.0278 ***	0.0049	0.0182 **	0.0579 ***	-0.0048	-0.0110 *
2011	-0.0176	-0.0263 ***	-0.0148	0.0054	0.0428 ***	0.0174	-0.0046
2014	-0.0071	-0.0299 ***	0.0089	0.0136	0.0655 ***	0.0317 *	-0.0003
Individual-dummies	yes	yes	yes	yes	yes	yes	yes
R^2	0.5571	0.5598	0.5421	0.5367	0.5621	0.5639	0.5546
DIC	-2659	-2858	-2597	-2884	-2679	-2504	-3316
Instrumental equation for income pc growth							
Constant	0.0145 ***	0.0144 ***	0.0143 ***	0.0144 ***	0.0143 ***	0.0144 ***	0.0144 ***
Income pc gr, lagged	0.3644 ***	0.3637 ***	0.3667 ***	0.3660 ***	0.3738 ***	0.3673 ***	0.3643 ***
a_{iv}	-0.7921	-0.3450	-0.7414	-0.3697	-1.7248 ***	-1.1430 **	-0.4788 **
R^2	0.5330	0.5306	0.5309	0.5315	0.5353	0.5329	0.5309
PIP EU	0.1373	0.0162	0.0134	0.2082	0.0141	0.0071	0.0109
PIP OECD	0.0105	0.0197	0.0102	0.0144	0.0138	0.0134	0.9483
PIP Annex I	0.9780	0.0164	0.0053	0.0076	0.0110	0.9891	0.3057
Half-life	2.6	2.6	3.1	3.0	3.4	3.4	4.0
Half-life OECD	-	-	-	-	-	-	6.0
Half-life Annex I	2.3	-	-	-	-	2.9	-
ν	4	3	3	4	4	4	3
N	390	390	390	390	390	390	390

Note: * CI 90%, ** CI 95%, *** CI 99%. Definitions and further information are provided in the note to Table A.5.

Table A.7: Detailed results for individual-specific convergence of CH_4 production per value added

	agriculture	livestock	energy	manufacturing	services	transport	public admin.
**Individual-specific convergence: CH_4 production per value added**							
Constant	0.0619	0.2740 *	0.0169	0.1235	0.0623	0.0195	0.1745
Ln(emissions)	-0.2218 ***	-0.2102 ***	-0.2358 ***	-0.1925 ***	-0.2106 ***	-0.1739 ***	-0.1873 ***
Ln(emissions) EU	-0.0002	-0.0042	0.0004	-0.0002	0.0015	0.0022	-0.0004
Ln(emissions) OECD	-0.0005	0.0002	0.0393	0.0003	-0.0001	-0.0005	0.0404
Ln(emissions) Annex I	-0.0223 ***	0.0019	-0.0001	0.0004	0.0002	-0.0007	0.0006
Ln(income pc)	-0.0529 *	-0.0103	0.0078	-0.0801 **	-0.1066 *	-0.0601 *	-0.0334
Income pc growth	-0.4692	-1.2311 **	0.5570	-0.2868	-1.5272 *	-0.1067	-1.2120 **
Ln(pop. density)	-0.0619	0.0161	-0.0935	-0.0131	-0.2152 ***	-0.0153	-0.0368
Openness	0.0000	-0.0005 **	-0.0001	-0.0001	0.0016 ***	0.0001	-0.0004 **
Political regime	0.0005	0.0034 **	0.0049 **	-0.0015	-0.0015	-0.0011	0.0000
Nuclear %	-0.0025 **	-0.0008	-0.0028 *	-0.0004	0.0002	0.0005	0.0005
Fossil fuels %	-0.0020 **	-0.0006	-0.0001	-0.0009	0.0002	0.0003	0.0010
Fossil rents	-0.0032	-0.0002	-0.0063 *	0.0055	0.0080 *	0.0055 *	0.0020
VA cattle	-0.0083	0.0105 *	0.0031	0.0102	0.0004	0.0069	0.0093
VA energy	-0.0022	0.0010	0.0016	-0.0029	0.0023	0.0023	-0.0003
VA manufacturing	0.0042 *	0.0036 **	0.0030	0.0047	0.0025	0.0015	0.0019
VA services	0.0030	0.0004	0.0017	0.0006	0.0040	0.0024	0.0010
VA transport	-0.0002	-0.0002	-0.0033	-0.0056 *	0.0031	0.0108 **	0.0023
VA public admin.	0.0010	-0.0004	-0.0002	0.0024	0.0025	0.0005	0.0053 **
2004	0.0613 ***	0.0265 **	-0.0253	0.0165	-0.0570 ***	-0.0109	-0.0269 ***
2007	0.0248	0.0010	-0.0469 **	-0.0006	-0.0661 ***	-0.0309 *	-0.0226 *
2011	0.0601 ***	-0.0214 *	-0.0467 **	-0.0733 ***	0.0545 **	-0.0004	-0.0527 ***
2014	0.0350 *	-0.0407 ***	-0.0619 ***	-0.0359 *	-0.0026	-0.0147	-0.0617 ***
Individual-dummies	yes	yes	yes	yes	yes	yes	yes
R^2	0.6357	0.8290	0.6330	0.5123	0.5102	0.4155	0.7014
DIC	-2426	-2620	-2319	-2426	-1971	-2345	-2677
Instrumental equation for income pc growth							
Constant	0.0146 ***	0.0146 ***	0.0145 ***	0.0145 ***	0.0142 ***	0.0144 ***	0.0144 ***
Income pc gr, lagged	0.3605 ***	0.3630 ***	0.3626 ***	0.3628 ***	0.3694 ***	0.3647 ***	0.3642 ***
a_{iv}	-0.3037	0.6359	-0.5029	-0.0775	0.6943	-0.2579	0.1845
R^2	0.5321	0.5330	0.5311	0.5313	0.5311	0.5300	0.5308
PIP EU	0.0092	0.1070	0.0233	0.0078	0.0068	0.1086	0.0279
PIP OECD	0.0229	0.0370	0.6329	0.0201	0.0108	0.0295	0.7294
PIP Annex I	0.9904	0.0170	0.0288	0.0053	0.0047	0.0197	0.0170
Half-life	2.8	2.9	2.6	3.2	2.9	3.6	3.3
Half-life Annex I	2.5	-	-	-	-	-	-
ν	4	4	4	4	3	3	3
N	390	390	390	390	390	390	390

Note: * CI 90%, ** CI 95%, *** CI 99%. Definitions and further information are provided in the note to Table A.5.

Table A.8: Detailed results for individual-specific convergence of CH_4 consumption per value added

	agriculture		livestock		energy		manufacturing		services		transport		public admin.	
**Individual-specific convergence: CH_4 consumption per value added**														
Constant	-0.0341		0.0815		-0.0065		0.0977		-0.0535		-0.0130		0.1124	
Ln(emissions)	-0.0705	***	-0.0403	***	-0.1124	***	-0.0320	***	-0.0129	***	-0.0390	***	-0.1070	***
Ln(emissions) EU	0.0012		0.0153		0.0030		-0.0003		0.0058		-0.0003		-0.0004	
Ln(emissions) OECD	0.0001		-0.0014		0.0016		0.0028		0.0006		0.0019		-0.0001	
Ln(emissions) Annex I	-0.0072		-0.0011		-0.0015		-0.0015		-0.0003		-0.0003		0.0007	
Ln(income pc)	0.0123		0.0409		0.0515	*	-0.0614	***	0.0362		-0.0327		-0.0243	
Income pc growth	0.2971		-0.3109		1.8663	***	0.4292		-0.2917		0.3675		-0.2821	
Ln(pop. density)	-0.1856	***	-0.0599		-0.1044		-0.0003		-0.0909		-0.0087		0.0198	
Openness	0.0000		-0.0001		0.0002		0.0001		-0.0005	*	-0.0003		-0.0005	***
Political regime	-0.0007		0.0015		0.0067	**	-0.0024		-0.0018		-0.0010		-0.0001	
Nuclear %	0.0003		-0.0001		-0.0029	*	-0.0005		0.0002		0.0009		0.0003	
Fossil fuels %	-0.0004		0.0000		-0.0022	*	0.0003		-0.0001		0.0007		0.0005	
Fossil rents	0.0050		0.0033		0.0003		-0.0003		0.0027		0.0068	***	0.0008	
VA cattle	0.0155	**	0.0096		-0.0026		0.0013		-0.0014		0.0038		0.0043	
VA energy	0.0037		-0.0067	***	0.0028		0.0021		0.0005		0.0018		-0.0027	*
VA manufacturing	0.0068	***	0.0019		-0.0017		0.0052	***	-0.0013		0.0016		-0.0007	
VA services	0.0044	**	-0.0030		-0.0023		0.0044	***	0.0011		0.0023		-0.0011	
VA transport	0.0057	*	-0.0062	**	-0.0048		0.0006		0.0034		0.0103	***	-0.0001	
VA public admin.	0.0054	**	-0.0025		0.0016		0.0027	*	0.0006		0.0023		0.0011	
2004	0.1123	***	0.0131		0.0509	***	0.0186		0.0110		-0.0080		-0.0136	*
2007	-0.0077		-0.0032		-0.0038		-0.0063		-0.0113		-0.0447	***	-0.0049	
2011	-0.0034		-0.0096		-0.0047		-0.0213	*	-0.0192		-0.0180		-0.0269	***
2014	0.0259		-0.0056		-0.0066		-0.0021		0.0084		-0.0107		-0.0221	*
Individual-dummies	yes		yes		yes		yes		yes		yes		yes	
R^2	0.4385		0.4553		0.4332		0.4344		0.4183		0.4170		0.6614	
DIC	-2481		-2609		-2197		-2820		-2591		-2700		-2901	
Instrumental equation for income pc growth														
Constant	0.0144	***	0.0146	***	0.0143	***	0.0144	***	0.0147	***	0.0146	***	0.0146	***
Income pc gr, lagged	0.3655	***	0.3609	***	0.3708	***	0.3656	***	0.3587	***	0.3610	***	0.3618	***
a_{iv}	-0.6363		-0.1707		-1.2085	*	-1.2867	***	-0.4985		-0.6842	*	-0.6379	*
R^2	0.5319		0.5314		0.5323		0.5314		0.5326		0.5311		0.5315	
PIP EU	0.0061		0.3557		0.1062		0.0061		0.5782		0.0057		0.0419	
PIP OECD	0.0180		0.0292		0.0309		0.0055		0.0033		0.0219		0.0478	
PIP Annex I	0.4829		0.0116		0.0270		0.1977		0.0237		0.0089		0.0069	
Half-life	9.5		16.9		5.8		21.3		53.4		17.4		6.1	
ν	4		4		3		3		5		4		4	
N	390		390		390		390		390		390		390	

Note: * CI 90%, ** CI 95%, *** CI 99%. Definitions and further information are provided in the note to Table A.5.

A.3.2 International convergence

Table A.9: Detailed results for international convergence of CH_4 production per capita

	agriculture		livestock		energy		manufacturing		services		transport		public admin.	
**International convergence: CH_4 production per capita**														
Constant	- 0.0405		- 0.0049		0.0787		- 0.0221		- 0.1713	*	- 0.0471		0.0087	
Ln(emissions)	- 0.0037	*	- 0.0007		- 0.0030		- 0.0137	***	- 0.0287	***	- 0.0054		- 0.0004	
Ln(emissions) EU	- 0.0017		- 0.0014		0.0017		0.0018		0.0017		- 0.0003		- 0.0007	
Ln(emissions) OECD	- 0.0018		- 0.0003		- 0.0006		- 0.0009		- 0.0007		0.0013		0.0014	
Ln(emissions) Annex I	- 0.0025		0.0021		- 0.0002		0.0010		- 0.0012		0.0006		- 0.0011	
Ln(income pc)	- 0.0004		0.0012		- 0.0032		0.0086		- 0.0070		0.0049		- 0.0050	
Income pc growth	1.0143	***	- 0.0960		0.6370	**	- 0.4111		0.4863		0.7984	**	0.2726	**
Ln(pop. density)	- 0.0021		0.0000		- 0.0031		0.0003		- 0.0023		0.0065	**	- 0.0007	
Openness	0.0000		- 0.0001	**	0.0000		- 0.0001		0.0002		0.0000		0.0001	
Political regime	0.0002		- 0.0008	***	- 0.0004		0.0002		0.0025		0.0008		0.0001	
Nuclear %	- 0.0001		- 0.0001		- 0.0001		0.0001		0.0001		0.0001		0.0001	
Fossil fuels %	- 0.0001		0.0000		0.0000		0.0000		- 0.0001		- 0.0002		0.0000	
Fossil rents	- 0.0005		- 0.0004		- 0.0001		0.0017		- 0.0035	*	0.0002		0.0004	
VA cattle	- 0.0012		0.0010		- 0.0023		0.0000		- 0.0027		- 0.0005		0.0009	
VA energy	- 0.0004		- 0.0003		- 0.0011		- 0.0014		0.0016		0.0001		0.0003	
VA manufacturing	- 0.0004		- 0.0001		- 0.0007		0.0001		- 0.0006		- 0.0009		0.0005	
VA services	0.0001		- 0.0001		- 0.0008		- 0.0011		- 0.0006		- 0.0008		0.0001	
VA transport	0.0001		- 0.0005		- 0.0009		0.0002		- 0.0009		0.0012		0.0010	**
VA public admin.	- 0.0013		- 0.0003		0.0000		- 0.0002		- 0.0010		- 0.0010		- 0.0001	
2004	0.0499	***	0.0096	***	0.0284	***	- 0.0342	**	0.1032	***	- 0.0131		0.0010	
2007	0.0397	***	0.0094	***	0.0159	*	- 0.0525	***	0.0921	***	- 0.0184		- 0.0019	
2011	0.0839	***	0.0094	***	0.0074		- 0.1016	***	0.2056	***	0.0139		0.0014	
2014	0.0334	***	0.0128	***	0.0184	**	- 0.0204		0.0893	***	0.0003		0.0004	
Individual-dummies	no		no		no		no		no		no		no	
R^2	0.1337		0.0114		0.0536		0.1322		0.0924		0.0481		0.0746	
DIC	- 2694		- 3552		- 2815		- 2644		- 2082		- 2665		- 3562	
Instrumental equation for income pc growth														
Constant	0.0143	***	0.0144	***	0.0144	***	0.0144	***	0.0143	***	0.0144	***	0.0144	***
Income pc gr, lagged	0.3678	***	0.3657	***	0.3653	***	0.3657	***	0.3663	***	0.3653	***	0.3642	***
a_{iv}	- 1.2569	***	0.1859		- 0.2730		1.2192	**	- 0.2760		- 0.2569		- 0.1724	
R^2	0.5312		0.5304		0.5301		0.5319		0.5302		0.5301		0.5300	
PIP EU	0.0058		0.0039		0.0084		0.0028		0.0060		0.0093		0.0338	
PIP OECD	0.0030		0.0021		0.0057		0.0064		0.0041		0.0033		0.3468	
PIP Annex I	0.0201		0.0032		0.0098		0.0531		0.0051		0.0079		0.0061	
Half-life	187.0		-		-		50.2		23.8		-		-	
ν	3		3		3		4		3		3		3	
N	390		390		390		390		390		390		390	

Note: * CI 90%, ** CI 95%, *** CI 99%. Definitions and further information are provided in the note to Table A.5.

Table A.10: Detailed results for international convergence of CH_4 consumption per capita

	agriculture		livestock		energy		manufacturing		services		transport		public admin.	
**International convergence: CH_4 consumption per capita**														
Constant	- 0.2413	***	0.0255		- 0.0434		- 0.4231	***	- 0.5652	***	- 1.0239	***	- 0.0915	***
Ln(emissions)	- 0.0371	***	- 0.0181	***	- 0.0226	***	- 0.0562	***	- 0.0501	***	- 0.0821	***	- 0.0215	***
Ln(emissions) EU	- 0.0002		- 0.0004		0.0002		- 0.0004		- 0.0009		0.0018		0.0000	
Ln(emissions) OECD	- 0.0011		0.0002		0.0000		- 0.0017		0.0003		0.0021		0.0002	
Ln(emissions) Annex I	- 0.0012		0.0001		- 0.0007		0.0002		0.0001		- 0.0043		- 0.0011	
Ln(income pc)	0.0164	*	0.0009		0.0188	*	0.0410	***	0.0555	***	0.0796	***	0.0068	
Income pc growth	1.1193	**	- 0.2941		0.9276	*	0.4823		1.3760	***	1.7038	***	0.6050	***
Ln(pop. density)	0.0076	**	- 0.0054	*	- 0.0026		- 0.0060	**	- 0.0009		- 0.0019		- 0.0067	***
Openness	- 0.0001		- 0.0001		0.0003	**	0.0001	**	0.0001		0.0001		0.0001	**
Political regime	0.0007		- 0.0011		0.0006		- 0.0016	*	- 0.0008		0.0040	***	0.0001	
Nuclear %	0.0000		- 0.0003		0.0003		0.0002		- 0.0005	**	- 0.0003		0.0001	
Fossil fuels %	0.0001		- 0.0003	**	0.0003		0.0000		- 0.0002		- 0.0001		0.0001	
Fossil rents	- 0.0008		0.0001		0.0008		- 0.0005		- 0.0015		0.0042	**	0.0000	
VA cattle	- 0.0012		- 0.0015		- 0.0040		- 0.0048	**	- 0.0009		- 0.0026		0.0002	
VA energy	0.0003		- 0.0005		- 0.0031	*	- 0.0009		0.0003		0.0004		0.0000	
VA manufacturing	- 0.0021	*	0.0003		- 0.0040	***	- 0.0011		- 0.0016		- 0.0005		0.0001	
VA services	- 0.0012		0.0001		- 0.0028	**	- 0.0006		- 0.0014		- 0.0007		0.0001	
VA transport	- 0.0011		- 0.0010		- 0.0018		- 0.0011		- 0.0009		0.0025		0.0013	**
VA public admin.	0.0005		- 0.0007		0.0007		0.0002		0.0006		- 0.0002		- 0.0007	
2004	0.0315	**	- 0.0025		- 0.0263	*	0.0343	***	0.0762	***	0.0313	**	0.0013	
2007	0.0038		0.0026		- 0.0483	***	0.0164	*	0.0280	**	- 0.0264	*	- 0.0063	
2011	0.0310	**	0.0082		- 0.0550	***	0.0003		0.0053		- 0.0105		- 0.0017	
2014	0.0266	**	0.0030		- 0.0290	**	0.0015		0.0180		- 0.0216		- 0.0003	
Individual-dummies	no		no		no		no		no		no		no	
R^2	0.1583		0.0996		0.1099		0.2466		0.2892		0.2973		0.0988	
DIC	-2622		-2840		-2488		-2914		-2712		-2546		-3383	
Instrumental equation for income pc growth														
Constant	0.0146	***	0.0144	***	0.0144	***	0.0145	***	0.0146	***	0.0145	***	0.0144	***
Income pc gr, lagged	0.3643	***	0.3653	***	0.3666	***	0.3626	***	0.3627	***	0.3657	***	0.3639	***
a_{iv}	- 0.7070		0.4890		- 0.5829		0.5381		- 0.8564	*	- 0.8495	*	- 0.4427	**
R^2	0.5331		0.5302		0.5314		0.5309		0.5338		0.5325		0.5304	
PIP EU	0.0100		0.0061		0.0066		0.0125		0.0071		0.0144		0.0331	
PIP OECD	0.0053		0.0070		0.0050		0.0111		0.0090		0.0053		0.0087	
PIP Annex I	0.0397		0.0056		0.0061		0.0041		0.0057		0.2406		0.0101	
Half-life	18.3		37.9		30.3		12.0		13.5		8.1		31.9	
ν	4		3		3		4		5		4		3	
N	390		390		390		390		390		390		390	

Note: * CI 90%, ** CI 95%, *** CI 99%. Definitions and further information are provided in the note to Table A.5.

Table A.11: Detailed results for international convergence of CH_4 production per value added

	agriculture		livestock		energy		manufacturing		services		transport		public admin.	
	**International convergence: CH_4 production per value added**													
Constant	0.0566		0.1634		- 0.1575	*	0.2440	**	0.1483		0.2072	**	- 0.1092	
Ln(emissions)	- 0.0107	***	- 0.0211	***	- 0.0123	***	- 0.0227	***	- 0.0239	***	- 0.0152	***	- 0.0039	
Ln(emissions) EU	- 0.0004		- 0.0008		0.0027		0.0020		- 0.0017		0.0013		0.0001	
Ln(emissions) OECD	- 0.0013		0.0001		- 0.0004		0.0002		- 0.0009		0.0008		0.0220	*
Ln(emissions) Annex I	0.0004		0.0002		0.0009		- 0.0008		0.0002		0.0064		0.0003	
Ln(income pc)	0.0147		0.0001		0.0004		- 0.0076		- 0.0458	**	- 0.0068		0.0006	
Income pc growth	1.0057	*	- 0.3009		0.7413		- 1.4113	**	- 0.4106		- 0.7394		- 0.0449	
Ln(pop. density)	- 0.0086	*	- 0.0091	**	0.0009		- 0.0094	*	- 0.0078		- 0.0090		0.0026	
Openness	- 0.0002		- 0.0001		- 0.0001		- 0.0001		0.0001		0.0001		- 0.0001	
Political regime	0.0019		0.0016		0.0010		0.0020		0.0013		0.0014		- 0.0010	
Nuclear %	0.0003		0.0002		- 0.0001		0.0002		0.0003		0.0003		0.0002	
Fossil fuels %	0.0001		0.0001		0.0001		0.0000		0.0000		0.0000		0.0000	
Fossil rents	0.0034	**	0.0040	***	- 0.0082	***	0.0071	***	- 0.0003		0.0034	*	0.0002	
VA cattle	- 0.0026		0.0059	**	-		- 0.0006		- 0.0019		- 0.0053		0.0017	
VA energy	- 0.0067	***	- 0.0049	***	0.0083	***	- 0.0056	***	0.0004		- 0.0028		- 0.0005	
VA manufacturing	- 0.0020		- 0.0003		0.0004		0.0010		0.0000		- 0.0011		- 0.0002	
VA services	- 0.0030	**	- 0.0016		0.0005		- 0.0031	**	0.0013		- 0.0017		- 0.0006	
VA transport	- 0.0028		- 0.0032	*	- 0.0033		0.0007		- 0.0010		0.0032		0.0022	
VA public admin.	- 0.0054	***	- 0.0025	*	- 0.0012		- 0.0019		- 0.0018		- 0.0027	*	0.0064	***
2004	0.1548	***	0.0550	***	0.0566	***	- 0.0195		0.0537	**	- 0.0092		- 0.0073	
2007	0.0799	***	0.0154		0.0527	***	- 0.0363	**	0.0808	***	- 0.0047		0.0230	*
2011	0.1075	***	- 0.0003		0.0710	***	- 0.0939	***	0.2168	***	0.0217		0.0059	
2014	0.0584	***	0.0109		0.0717	***	- 0.0068		0.0888	***	- 0.0093		0.0207	*
Individual-dummies	no		no		no		no		no		no		no	
R^2	0.2741		0.1354		0.1748		0.1773		0.1088		0.0556		0.2117	
DIC	-2377		-2585		-2336		-2416		-2016		-2341		-2665	
Instrumental equation for income pc growth														
Constant	0.0144	***	0.0145	***	0.0143	***	0.0144	***	0.0143	***	0.0143	***	0.0144	***
Income pc gr, lagged	0.3700	***	0.3629	***	0.3668	***	0.3668	***	0.3666	***	0.3659	***	0.3652	***
a_{iv}	- 2.0212	***	- 0.5782		- 0.7867		1.0637	*	- 0.4524		- 0.2256		- 0.9588	**
R^2	0.5342		0.5316		0.5308		0.5320		0.5304		0.5304		0.5309	
PIP EU	0.0099		0.0195		0.0154		0.0048		0.0045		0.0200		0.0058	
PIP OECD	0.0062		0.0239		0.0072		0.0148		0.0036		0.0090		0.9295	
PIP Annex I	0.0244		0.0102		0.0098		0.0085		0.0032		0.4782		0.0250	
Half-life	64.4		32.5		56.0		30.2		28.7		45.3		-	
Half-life OECD	-		-		-		-		-		-		-38.6	
ν	4		4		3		4		3		3		3	
N	390		390		390		390		390		390		390	

Note: * CI 90%, ** CI 95%, *** CI 99%. Definitions and further information are provided in the note to Table A.5.

Table A.12: Detailed results for international convergence of CH_4 consumption per value added

	agriculture		livestock		energy		manufacturing		services		transport		public admin.	
**International convergence: CH_4 consumption per value added**														
Constant	- 0.1188	*	0.1527	*	- 0.0580		- 0.0674		0.0622		0.1056	**	- 0.0802	
Ln(emissions)	- 0.0032		- 0.0164	***	- 0.0104	**	- 0.0012		- 0.0021		- 0.0115	***	- 0.0007	
Ln(emissions) EU	0.0026		- 0.0003		- 0.0013		0.0002		- 0.0025		0.0008		- 0.0008	
Ln(emissions) OECD	0.0007		0.0008		- 0.0002		- 0.0026		- 0.0017		- 0.0015		0.0041	
Ln(emissions) Annex I	0.0012		0.0004		- 0.0009		0.0000		- 0.0009		- 0.0013		0.0002	
Ln(income pc)	0.0129		0.0037		0.0165		- 0.0135	**	- 0.0077		- 0.0072		0.0006	
Income pc growth	0.6236		- 0.1654		1.1656	**	0.0095		- 0.0278		0.3172		0.2812	
Ln(pop. density)	- 0.0023		- 0.0060	*	0.0033		- 0.0043	**	- 0.0037		- 0.0079	***	- 0.0003	
Openness	- 0.0001		0.0000		0.0001		0.0000		- 0.0001		0.0001		0.0000	
Political regime	0.0017	*	0.0012		0.0038	***	- 0.0009		- 0.0009		0.0003		- 0.0006	
Nuclear %	0.0001		- 0.0002		- 0.0005		0.0000		0.0000		0.0000		0.0002	
Fossil fuels %	0.0001		- 0.0002		- 0.0002		0.0001		- 0.0001		0.0001		0.0000	
Fossil rents	0.0022		0.0038	***	- 0.0052	**	0.0005		0.0001		0.0020	*	0.0002	
VA cattle	0.0009		- 0.0005		- 0.0082	**	- 0.0006		- 0.0032		- 0.0041	**	0.0016	
VA energy	- 0.0027	**	- 0.0048	***	0.0037		0.0005		- 0.0006		- 0.0013		- 0.0007	
VA manufacturing	- 0.0003		- 0.0003		- 0.0037	**	0.0030	***	- 0.0002		- 0.0002		- 0.0003	
VA services	- 0.0010		- 0.0020	**	- 0.0034	**	0.0021	***	0.0002		- 0.0004		- 0.0005	
VA transport	- 0.0006		- 0.0026	*	- 0.0040	*	0.0029	***	0.0015		0.0020		0.0018	
VA public admin.	- 0.0004		- 0.0012		- 0.0006		0.0019	**	- 0.0003		- 0.0005		0.0031	**
2004	0.1434	***	0.0183	*	0.1028	***	0.0141	*	0.0164		- 0.0133		- 0.0022	
2007	0.0136		- 0.0131		0.0651	***	- 0.0079		0.0086		- 0.0429	***	0.0178	**
2011	0.0085		- 0.0214	**	0.0720	***	- 0.0207	***	- 0.0009		- 0.0239	**	0.0043	
2014	0.0254	**	- 0.0102		0.0797	***	0.0033		0.0195	**	- 0.0231	**	0.0239	***
Individual-dummies	no		no		no		no		no		no		no	
R^2	0.3101		0.2694		0.1805		0.2803		0.2369		0.1646		0.3063	
DIC	-2644		-2749		-2301		-3005		-2781		-2844		-2915	
Instrumental equation for income pc growth														
Constant	0.0144	***	0.0146	***	0.0143	***	0.0144	***	0.0147	***	0.0145	***	0.0144	***
Income pc gr, lagged	0.3658	***	0.3624	***	0.3681	***	0.3649	***	0.3588	***	0.3619	***	0.3662	***
a_{iv}	- 1.2754	***	- 0.4804		- 0.8145		- 0.8734	***	- 0.8619	**	- 0.8045	**	- 1.1868	***
R^2	0.5320		0.5320		0.5316		0.5309		0.5321		0.5310		0.5313	
PIP EU	0.0097		0.0067		0.0149		0.0021		0.0009		0.0024		0.0050	
PIP OECD	0.0040		0.0257		0.0111		0.0040		0.0011		0.0032		0.3341	
PIP Annex I	0.0342		0.0220		0.0070		0.0213		0.0009		0.0249		0.0070	
Half-life	-		41.9		66.3		-		-		59.9		-	
ν	4		4		3		3		4		4		3	
N	390		390		390		390		390		390		390	

Note: * CI 90%, ** CI 95%, *** CI 99%. Definitions and further information are provided in the note to Table A.5.

A.4 Robustness of the results for different values of ν

We performed several robustness checks to analyze the sensitivity of the results from economy-wide regressions to the degree of heteroscedasticity. These checks consisted in (i) specifying alternative values for the hyperprior of the hyperparameter ν (see Fernández-Amador et al., 2019, for details on the parameters), and (ii) imposing different values for ν exogenously. In the first experiment, alternative values of the hyperprior for ν did neither affect the estimated value of ν nor the results concerning the existence of convergence reported in the main text. Therefore, we conclude that the estimates of the degree of heteroscedasticity are rather robust.

In the second experiment, we exogenously fixed the parameter ν, such that $\nu = \{10, 20, 40\}$. The results are summarized in Table A.13, where we report the estimated values of the convergence parameter, β, and the implied half-lives for convergence towards international steady states conditional on the control variables for economy-wide emissions. The results suggest that the estimates reported in the main text are robust to a wide range of degrees of heteroscedasticity. Specifically, the results remain robust, for most emission inventories, for values of ν ranging up to $\nu = 40$. For production-based emissions per value added, the estimates of the heteroscedastic model are not qualitatively affected for $\nu = 10$, but there is some (marginal) evidence for convergence for higher values of ν. In general, the speed of convergence is estimated to be faster as the degree of heteroscedasticity is restricted to be lower (as the value of ν increases).

Table A.13: International convergence—Robustness to different values of ν

		(1) CH_4 pc prod.	(2) CH_4 pc cons.	(3) CH_4 va prod.	(4) CH_4 va cons.
$\nu = 10$	Ln(emissions)	-0.0026	-0.0204 ***	-0.0055	-0.0026
	Half-life	266	34	126	266
$\nu = 20$	Ln(emissions)	-0.0036	-0.0231 ***	-0.0066 *	-0.0034
	Half-life	192	30	105	204
$\nu = 40$	Ln(emissions)	-0.0051	-0.027 ***	-0.0082 **	-0.0047
	Half-life	136	25	84	147

Note: * CI 90%, ** CI 95%, *** CI 99%.
